# Materials synthesis at terapascal static pressures

**DOI:** 10.1038/s41586-022-04550-2

**Published:** 2022-05-11

**Authors:** Leonid Dubrovinsky, Saiana Khandarkhaeva, Timofey Fedotenko, Dominique Laniel, Maxim Bykov, Carlotta Giacobbe, Eleanor Lawrence Bright, Pavel Sedmak, Stella Chariton, Vitali Prakapenka, Alena V. Ponomareva, Ekaterina A. Smirnova, Maxim P. Belov, Ferenc Tasnádi, Nina Shulumba, Florian Trybel, Igor A. Abrikosov, Natalia Dubrovinskaia

**Affiliations:** 1 Bayerisches Geoinstitut, University of Bayreuth, Bayreuth, Germany; 2 Material Physics and Technology at Extreme Conditions, Laboratory of Crystallography University of Bayreuth, Bayreuth, Germany; 3 Deutsches Elektronen-Synchrotron (DESY), Hamburg, Germany; 4 Institute of Inorganic Chemistry, University of Cologne, Cologne, Germany; 5 European Synchrotron Radiation Facility, Grenoble, France; 6 Center for Advanced Radiation Sources, The University of Chicago, Chicago, IL USA; 7 Materials Modeling and Development Laboratory, National University of Science and Technology “MISIS”, Moscow, Russia; 8 Theoretical Physics Division, Department of Physics, Chemistry and Biology (IFM), Linköping University, Linköping, Sweden

**Keywords:** Phase transitions and critical phenomena, Chemical physics, Solid-state chemistry, Condensed-matter physics

## Abstract

Theoretical modelling predicts very unusual structures and properties of materials at extreme pressure and temperature conditions^1,2^. Hitherto, their synthesis and investigation above 200 gigapascals have been hindered both by the technical complexity of ultrahigh-pressure experiments and by the absence of relevant in situ methods of materials analysis. Here we report on a methodology developed to enable experiments at static compression in the terapascal regime with laser heating. We apply this method to realize pressures of about 600 and 900 gigapascals in a laser-heated double-stage diamond anvil cell^3^, producing a rhenium–nitrogen alloy and achieving the synthesis of rhenium nitride Re_7_N_3_—which, as our theoretical analysis shows, is only stable under extreme compression. Full chemical and structural characterization of the materials, realized using synchrotron single-crystal X-ray diffraction on microcrystals in situ, demonstrates the capabilities of the methodology to extend high-pressure crystallography to the terapascal regime.

## Abstract (web summary)

Pressures of up to 900 gigapascals (9 million atmospheres) are achieved in a laser-heated double-stage diamond cell, enabling the synthesis of Re_7_N_3_, and materials characterization is performed in situ using single-crystal X-ray diffraction.

## Main

The state of matter is strongly affected by variations in chemical composition and external parameters such as pressure and temperature, enabling tuning of material properties. This gives rise to various phenomena relevant for a broad range of scientific disciplines and technological applications, from fundamental understanding of the Universe to targeted design of advanced materials. Compression is known to facilitate metal-to-insulator transitions^4^, superconductivity^5^ and new ‘super’ states of matter^6^. Recent developments in the diamond anvil cell technique, and, particularly, the invention of double-stage and toroidal diamond anvil cells (dsDACs and tDACs)^3,7,8^, have enabled breakthroughs in the synthesis of materials and the study of structure–property relationships at high and ultrahigh pressures. Very recent examples are the discovery of a new nitrogen allotrope^9^, bp-N, which resolved a puzzle in our understanding of the high-pressure behaviour of pnictogen family elements, and the synthesis of a plethora of novel transition metal nitrides and polynitrides^10–15^, including metal–inorganic frameworks^11,15^, which are a new class of compounds featuring open porous structures at megabar compression. Solving and refining the crystal structures of solids synthesized directly from elements in laser-heated conventional DACs^10–15^ at pressures as high as up to about two megabars^12,16^ became possible owing to the synergy of our expertise both in generating pressures of several megabars^3,17,18^ (for details see Supplementary Information section ‘Brief overview of the double-stage DAC (dsDAC) technique’) and in single-crystal X-ray diffraction (XRD) at ultrahigh pressures, which were pioneered a few years ago^19,20^. As the high-pressure high-temperature synthesis has become a well established technique for materials discovery, extending investigations to the TPa regime has long been desired.

Here we report a methodology for high-pressure high-temperature synthesis experiments that extends the limits of high-pressure crystallography to the terapascal range. To achieve the desired pressures, we combined toroidal^7,8^ and double-stage^3,17,18^ anvil designs. A rhenium–nitrogen alloy and rhenium nitride Re_7_N_3_ were synthesized in three different experiments in the Re–N system (Supplementary Table 1) in a laser-heated dsDAC. Their full structural and chemical characterization was performed in situ using single-crystal XRD.

**Extended Data Fig. 1:**
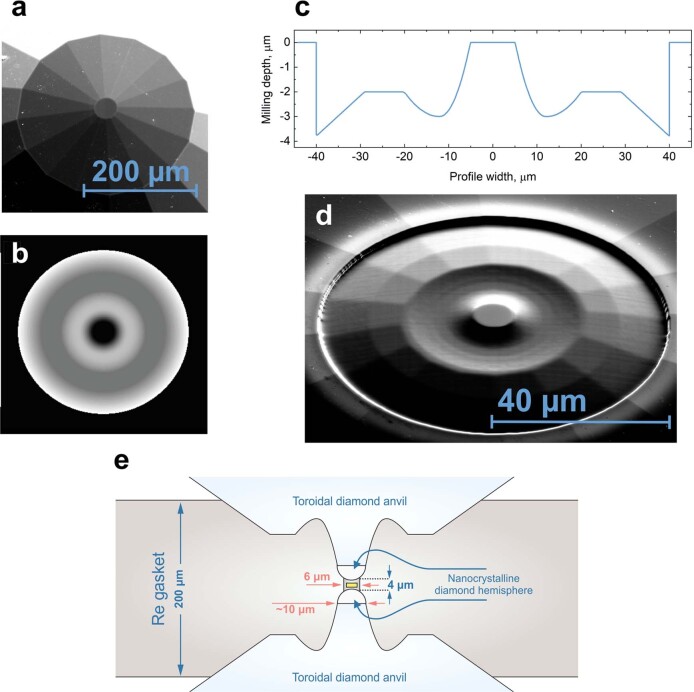
Toroidal profile produced by FIB milling on a culet of a conventional anvil. **a**, Original Boehler–Almax-type single-bevelled diamond anvils with 40-μm culets (before milling). **b**, Greyscale bitmap used for milling. **c**, The milling depth profile. **d**, Crafted toroidal culet. **e**, Cross-sectional schematic of the dsDAC assembly (not to scale): a gasket (grey) with the pressure chamber (dark grey; the sample is yellow) squeezed between the two toroidal diamond anvils (light blue) equipped with hemispheres of nanocrystalline diamond (white); the pressure chamber is of 6 μm in diameter and 4 μm in height. The gasket was prepared as follows: We used strips of a 200-μm thick Re foil. To make an indentation with a thickness of about 4 μm, we followed a many-step procedure. First, the Re foil was pre-indented to a thickness of ~20 μm using a pair of single-bevelled diamonds with 80-μm culets. Then, a hole about 30 μm in diameter was laser-drilled in the centre of the indentation, and the indentation was pressurized again between the same bevelled diamonds. This led to closing of the hole and a reduction in the thickness of the indentation. This procedure was repeated a few times until a thickness of about 7 μm was achieved. The gasket was mounted into a BX-90 DAC equipped with toroidal diamond anvil and indented by their miniature 10-μm culets to a thickness of ~4 μm. A hole (~6 μm in diameter) in the centre of the ~10-μm indentation was made using FIB or by tightly focused pulsed near-infrared laser to form a pressure chamber.

**Extended Data Fig. 2:**
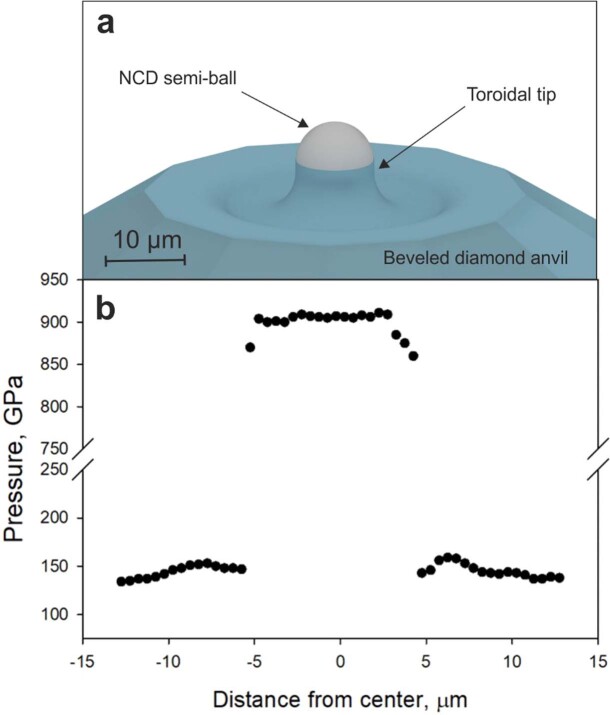
A schematic illustration of the experimental set-up. **a**, Three-dimensional presentation of a diamond anvil featuring a toroidal profile milled by FIB on the surface of a conventional Boehler–Almax-type single-bevelled diamond anvil with a 40-μm culet. A hemisphere of transparent nanocrystalline diamond (NCD)^17^, which was FIB-milled from a single ball with a diameter of 12 to 14 μm, was placed over the tip to realize a double-stage DAC design. Two anvils of this kind were forced together as shown in the scheme in Extended Data Fig. 1. **b**, Pressure profile along the cross-section through the centre of dsDAC #1 after pulsed laser-heating. Diffraction patterns were collected at each point with a step of 0.5 μm at the ID11 beamline at ESRF, and pressure was determined according to the equation of state from ref. ^27^ using the lattice parameters of Re found from powder diffraction data.

The dsDACs were prepared following the procedure outlined below. Conventional Boehler–Almax-type single-bevelled diamond anvils with 40-μm culets were milled by focused ion beam (FIB) in order to produce a toroidal profile on the surface of the culet and to shape a miniature culet of about 10 μm in diameter in its centre (Extended Data Fig. 1). As a gasket we used a strip of a 200-μm-thick Re foil, which was pre-indented in a few steps. The final indentation of 10 μm in diameter (made using anvils with the toroidal profile) had a thickness of about 4 μm (the indentation procedure is described in detail in the legend to Extended Data Fig. 1). A hole of approximately 6 μm in diameter was made in the centre of the indentation using FIB or by tightly focused pulsed near-infrared laser to form a pressure chamber. A schematic of the dsDAC assembly, mounted into a BX-90 DAC^21^ equipped with toroidal diamond anvils, is shown in Extended Data Fig. 1. To realize a dsDAC design, two transparent nanocrystalline diamond^17^ hemispheres, FIB-milled from a single ball with a diameter of 12 to 14 μm, were placed over the tip of the 10-μm culet (Extended Data Figs. 1, 2). The hemispheres were small enough to stick on the toroidal anvils, but in one case (dsDAC #2, Supplementary Table 1) paraffin wax was used to affix them. A few grains of a rhenium powder (99.995% purity, Merck) were placed into the pressure chamber, which was then filled with nitrogen (N_2_) at about 1.4 kbar using the high-pressure gas-loading set-up^22^ at Bayerisches Geoinstitut (BGI, Bayreuth, Germany), closed, and pressurized.

**Extended Data Fig. 3:**
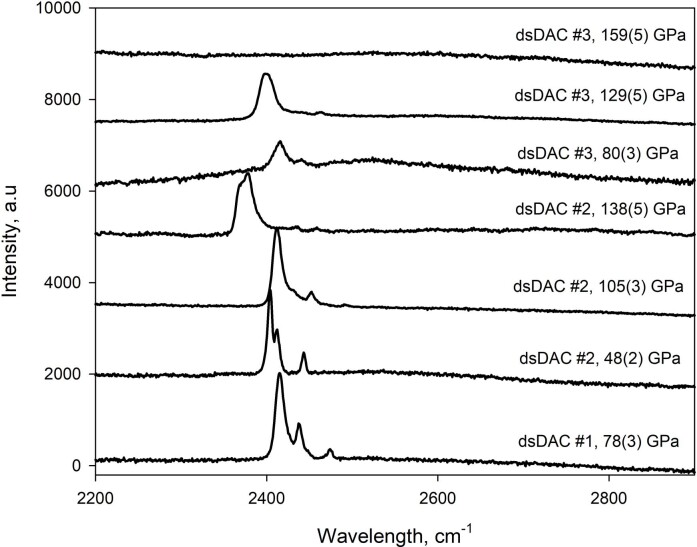
Raman spectra taken upon compression of the dsDAC after nitrogen gas loading featuring nitrogen vibron. At pressures above ~160 GPa the Raman signal of nitrogen becomes non-detectable. The pressures were determined according to ref. ^24^. a.u., arbitrary units.

**Extended Data Fig. 4:**
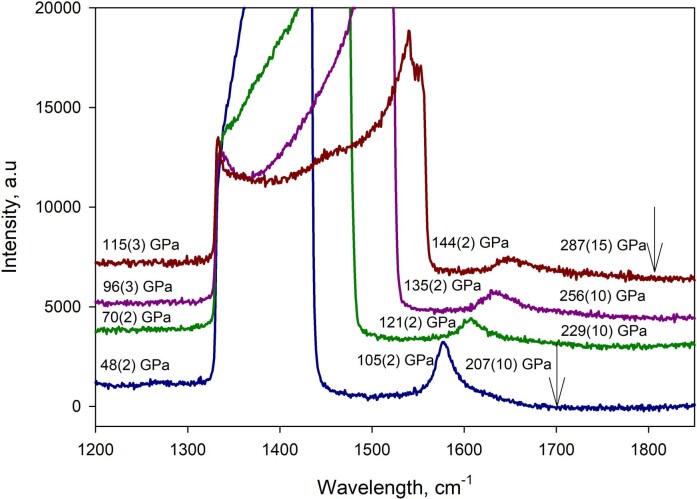
The evolution of Raman spectra of primary and secondary anvils upon compression of dsDAC #2. The pressure is determined from diamond line Raman shift^24^. The pressures on primary anvil are shown on the left. The peaks at ~1,600 cm^−1^ are the pressures on the body of the secondary anvil. The values on the right give estimates of pressures from the broadened Raman line of the secondary anvil (the arrows provide examples of positions found by analysis of the first derivatives of the spectra). Reliable determination of the pressure in the chamber from the Raman shift of the diamond line of the secondary nanocrystalline diamond anvils is not feasible.

After closing the cells in the pressure chambers, pressures were about 50 to 80 GPa (Extended Data Fig. 3); pressures on the primary anvils were below 10 GPa, as measured according to refs. ^23,24^. Our experience suggests that the cell should be pressurized quickly to approximately 40 GPa on the primary anvils to avoid loss of nitrogen. The presence of nitrogen can be monitored on N_2_ vibrons in the Raman spectra (Extended Data Fig. 3). However, N_2_ vibrons were not detectable above approximately 150 GPa (Extended Data Fig. 3) in the pressure chamber, because at such compression nitrogen becomes non-transparent and we can no longer detect the Raman signal. In dsDAC #2 we were able to observe the evolution of the Raman signal from the secondary anvil in parallel with that from the primary anvil upon pressurization (Extended Data Fig. 4). Huge stress on the secondary anvil is manifested in the large asymmetry of its corresponding Raman line, the high-frequency edge of which is difficult to determine reliably (Extended Data Fig. 4). Thus, it cannot be used for characterization of pressure in the sample chamber. (We also note that, as a rule, Raman spectra of nanocrystalline diamond are somewhat weak and broad).

In all dsDAC experiments described here, we followed the same protocol. After pressurization of the cells to about 120–140 GPa on the first-stage anvils^24^, the samples were laser-heated. The dsDACs #2 and #3 were heated by a pulsed laser (1-μs pulse duration, 25-kHz repetition rate, approximately 25 W at each side) at BGI using the set-up specially designed for ultrahigh pressures: the near-infrared (1,070 nm) laser beam is of less than 5 μm full-width at half-maximum (FWHM) in diameter and has an optical magnification of about 300×^25,26^. The entire pressure chamber of dsDAC #2 was heated at 2,900(200) K for about 3 min, and dsDAC #3 at 3,450(200) K for about 5 min. After laser-heating, the pressures on the primary anvils of dsDAC #2 and dsDAC #3 were about 100 GPa and 120 GPa, respectively.

**Extended Data Fig. 5:**
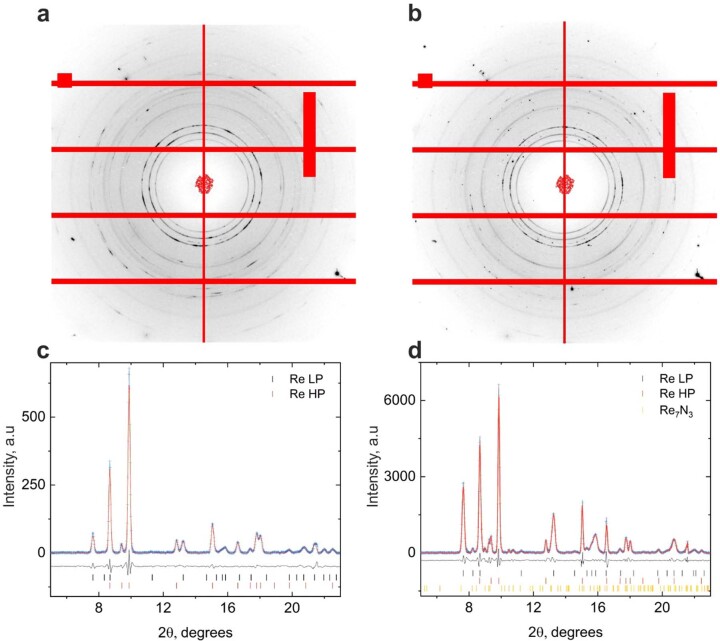
Examples of powder diffraction patterns collected from dsDAC #1. Data collected at 13-IDD (Advanced Photon Source, USA; beam FWHM, 3 × 3 μm^2^). **a**, The centre of the pressure chamber of the as-compressed ds-DAC. **c**, Re LP (low pressure): *a* = 2.5606(5) Å, *c* = 4.0588(12) Å and *V* = 23.047(7) Å^3^, that is, 149(3) GPa according to the equation of state from ref. ^27^, or 173(3) GPa according to ref. ^3^; Re HP (high pressure): *a* = 2.2214(3) Å, *c* = 3.5609(8) Å and *V* = 15.21(1) Å^3^, that is, 930(5) GPa^27^ or 1298(10) GPa^3^. **b**, After pulsed laser-heating at 2,200(200) K during 5 s. **d**, Re LP: *a* = 2.5577(3) Å, *c* = 4.1095(12) Å and *V* = 23.282(7) Å^3^, at 140(3) GPa^27^ or 162(3) GPa^3^; Re HP: *a* = 2.2297(2) Å, *c* = 3.5735(5) Å and *V* = 15.38(1) Å^3^, at 895(5) GPa^27^ or 1,250(10) GPa^3^; Re_7_N_3_: *a* = 6.3086(4) Å, *c* = 4.0048(7) Å and *V* = 138.04(4) Å^3^. Structural data for Re_7_N_3_ were taken from the results of single-crystal XRD data analysis.

The dsDAC #1 was heated at 13-IDD at GSECARS (Advanced Photon Source, USA) from both sides using a tightly focused near-infrared laser beam (FWHM of about 8 μm in diameter) in pulsed mode (1-μs pulse duration, 50-kHz repetition rate, approximately 20 W each side) for 5 s at a temperature of 2,200(200) K. Powder diffraction data acquired before laser-heating (Extended Data Fig. 5; at 13-IDD the X-ray beam had a FWHM of approximately 3 × 3 μm^2^) gave the following lattice parameters for Re: for the gasket, *a* = 2.5606(5) Å, *c* = 4.0588(12) Å, *V* = 23.047(7) Å^3^, and for the Re sample, *a* = 2.2214(3) Å, *c* = 3.5609(8) Å, *V* = 15.21(1) Å^3^. These parameters correspond to pressures of 149(3) GPa on the gasket and 930(5) GPa on the sample; the conservative values are given according to the equation of state from ref. ^27^ (Supplementary Table 1; the uncertainty in pressure corresponds to the statistical error in volume). X-ray powder diffraction patterns collected after laser-heating show that the positions of the diffraction lines of the Re gasket did not change within the accuracy of the measurements, and those from the Re sample changed very slightly (*a* = 2.2297(2) Å, *c* = 3.5735(5) Å, *V* = 15.38(1) Å^3^) corresponding to a pressure of 895(5) GPa (ref. ^27^).

After laser-heating for each dsDAC at 13-IDD at GSECARS, numerous diffraction spots were observed (Extended Data Fig. 5), indicating phase transformation(s) and/or chemical reaction(s) in the samples. However, interpreting the powder diffraction data turned out to be impossible, as the patterns were dominated by the diffraction lines from the gasket and untransformed Re, owing to the relatively large X-ray beam and a small sample size. Single-crystal diffraction data were of poor quality that precluded their analysis.

The dsDACs with temperature-quenched material were transported to ID11 at the European Synchrotron Radiation Facility (ESRF, Grenoble, France) and investigated using both powder and single-crystal XRD (see Methods). Despite the nominally small size of the X-ray beam, the reflections from the gasket were present even in the patterns collected from the centre of the sample chamber. Two-dimensional (2D) diffraction maps of still XRD images revealed powder diffraction of the Re gasket and untransformed material that enabled the analysis of the pressure distribution both within and around the sample (Extended Data Fig. 2). In dsDAC #1, for example, pressure at the sample/gasket boundary did not exceed approximately 160 GPa, and pressure at all points within the sample chamber was almost the same, of about 900 GPa (Extended Data Fig. 2). Our observations regarding the pressure distribution (Extended Data Fig. 3) in the sample chamber are consistent with those previously reported for toroidal-type anvils^7,8^ and give the pressure magnification factor (the ratio of the pressures on the primary and secondary anvils) of about 6, in accordance with previous publications on ds-DACs^17,28^.

**Fig. 1:**
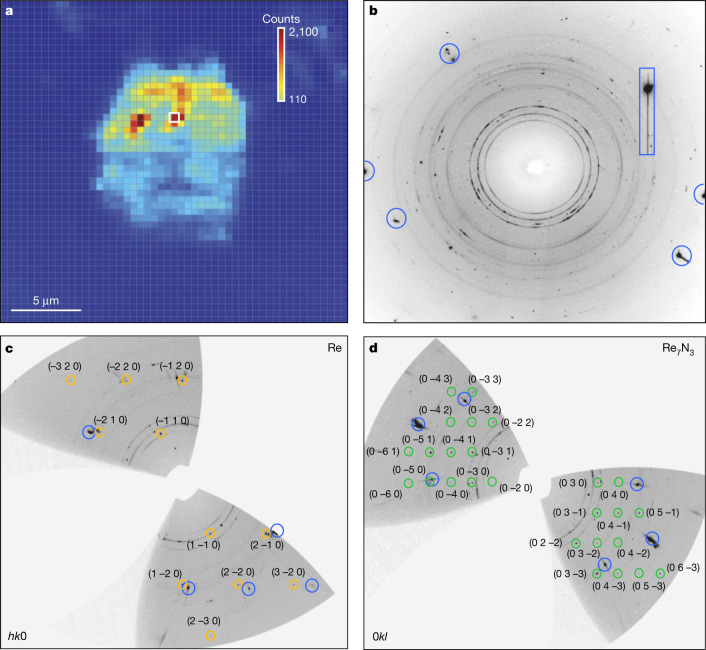
Results of XRD measurements on the sample of Re and N_2_ pulsed laser-heated in dsDAC #1. **a**, X-ray 2D map showing the distribution of different phases (recrystallized Re and Re_7_N_3_) in the pressure chamber of dsDAC #1. Each pixel on the map corresponds to a 2D XRD pattern collected at the Frelon 4M detector at the ID11 beamline at ESRF (beam size FWHM approximately 0.45 × 0.45 μm^2^, *λ* = 0.3099 Å). The map covers the whole pressure chamber (21.5 × 21.5 μm^2^, steps of 0.5 μm in both directions, 10-s acquisition time per frame). The total collection time was about 8 h. The colour intensity is proportional to the intensity of the following reflections: the (100) reflection of the Re gasket for the dark blue region; the (101) reflection of Re for the light blue region (inside the sample chamber); the inset colour bar corresponds to the sum of intensities of (202) and (420) reflections of Re_7_N_3_. **b**, Example of an as-collected diffraction image with diffraction lines and spots of Re (*a* = 2.2269(4) Å, *c* = 3.5702(15) Å) and Re_7_N_3_ (*a* = 6.2788(2) Å, *c* = 4.000(2) Å). The characteristic diffraction image shown in **b** is highlighted with a white rectangle in **a**. **c**, **d**, The reconstructed reciprocal lattice planes of Re (**c**) and Re_7_N_3_ (**d**). In **c**, **d**, the reflections of Re and Re_7_N_3_ are marked by yellow and green circles, respectively, and the corresponding *hkl* are given. Powder diffraction lines are due to the Re gasket and untransformed rhenium. In **b**–**d**, blue circles and the blue rectangle indicate parasitic reflections from diamond anvils.

**Fig. 2:**
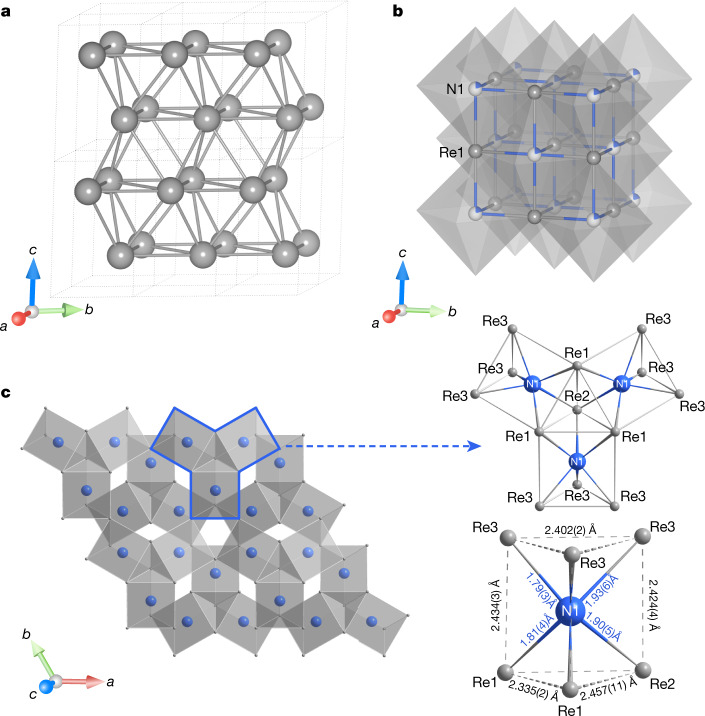
Crystal structures of the phases observed in laser-heated dsDACs. **a**, Hexagonal rhenium at 905(5) GPa in dsDAC #1 (*a* = 2.2269(4) Å, *c* = 3.5702(15) Å and *V* = 15.33(1) Å^3^). **b**, Cubic (B1 NaCl-type) rhenium–nitrogen solid solution ReN_0.2_ at 730(4) GPa (*a* = 3.3994(7) Å, *V* = 39.28(2) Å^3^). **c**, Hexagonal Re_7_N_3_ (*a* = 6.2788(2) Å, *c* = 4.000(2) Å and *V* = 136.53(11) Å^3^). In Re_7_N_3_, the structural units are NRe_6_ prisms with the nitrogen atom in the centre. Rhenium atoms are grey and nitrogen atoms are blue.

Apart from powder diffraction rings, the diffraction patterns collected at ID11 from certain locations in the sample area show numerous spots (Fig. 1). At these positions we collected single-crystal datasets upon rotation of the DAC around the *ω* axis from −38° to 38° with an angular step of 0.5° (Methods). For dsDAC #1, particularly, the analysis of single-crystal XRD data revealed the presence of domains of two phases (Supplementary Table 2). The first phase is hexagonal (space group *P*6_3_/*mmc*) with lattice parameters *a* = 2.2269(4) Å, *c* = 3.5702(15) Å and *V* = 15.33(1) Å^3^, as determined using 64 reflections. This was interpreted as Re (Figs. 1, 2) being under a pressure of 905(5) GPa (ref. ^27^). Within uncertainty, the *c*/*a* ratio (1.603(5)) coincides with that reported for pure Re at lower pressures^3,27^. The structure solution and refinement showed indeed that rhenium recrystallizes upon pulsed laser-heating (Fig. 2 and Supplementary Table 2), but is not contaminated by carbon or nitrogen (at least in the quantities that could be detectable from our XRD data).

The second phase found in the pressure chamber of the dsDAC #1 after heating is also hexagonal (space group *P*6_3_*mc*) and has lattice parameters *a* = 6.2788(2) Å, *c* = 4.000(2) Å and *V* = 136.53(11) Å^3^. On the basis of 394 independent reflections, the structure of this phase was solved and refined in isotropic approximation of atomic displacement parameters (Fig. 2 and Supplementary Table 2) to R_1_ = 5.7%. The chemical composition of the phase was refined as Re_7_N_3_. Considering the possibility of the reaction between rhenium and carbon from the anvils, we checked if the phase could be interpreted as carbide (Re_7_C_3_). In this case, however, the isotropic thermal parameter of carbon becomes negative, supporting the assignment of the atomic positions to nitrogen.

**Extended Data Fig. 6:**
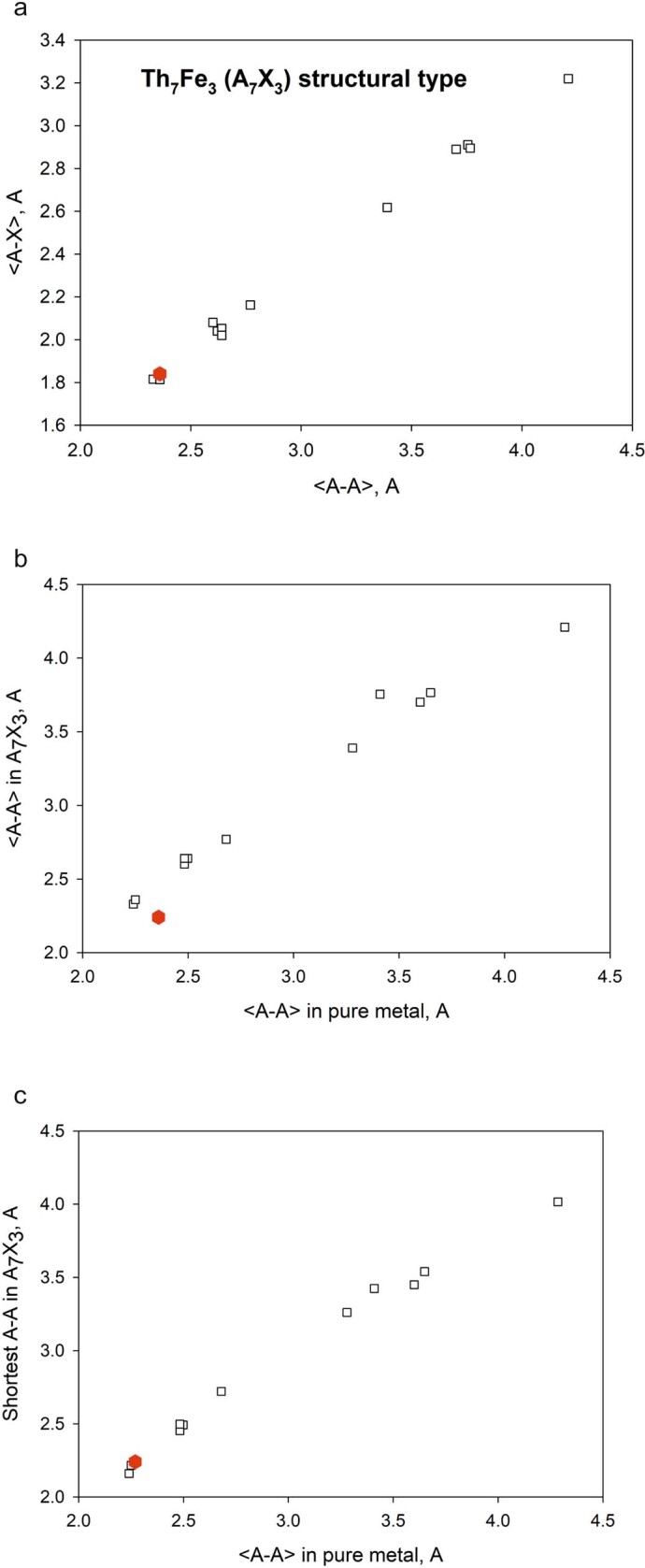
Interatomic distances in Th_7_Fe_3_ structured and some other selected *A*_7_*X*_3_ compounds. **a**, **b**, Comparison of shortest (**a**) and average between first neighbours (**b**) *A*–*A* distances in *A*_7_*X*_3_ compounds with metal–metal contacts in corresponding pure metals (*A*) at the same pressures. **c**, Correlation between average ⟨*X*–*A*⟩ distances in *XA*_6_ prisms and first neighbours ⟨*A*–*A*⟩ distances. The red hexagons correspond to Re_7_N_3_ as described in this work; data for orthorhombic Fe_7_C_3_ at 158 GPa are from ref. ^35^, data for predicted Fe_7_N_3_ at 150 GPa are from ref. ^32^, all other data are from ref. ^30^.

The structure units of Re_7_N_3_ are distorted NRe_6_ trigonal prisms (Fig. 2). Three prisms are connected through shared edges forming triads, which are stacked along the 6_3_ axis. Each triad is rotated by 60° with regard to upper and lower neighbours in the columns (Fig. 2). The columns are connected to each other by the common vertices of the prisms. Crystal structures built of combined triads of prisms are well known among carbides, borides, phosphides and nitrides^29^. Moreover, there are a number of binary compounds with the *A*_7_*X*_3_ stoichiometry (*A* and *X* are different chemical elements), and especially hexagonal ones with Th_7_Fe_3_-type structure (more than 70 entries in the ICSD database)^30^, the same as that of the Re_7_N_3_ compound. We noticed that in Re_7_N_3_, the shortest and average distances between the Re–Re nearest neighbours (approximately 2.28 Å and 2.37 Å, respectively) are just slightly longer than the Re–Re distances in metallic rhenium (about 2.23 Å), which is present in the pressure chamber along with the nitride. A comparison of the shortest and average distances between the closest *A*–*A* neighbours in the Th_7_Fe_3_-type structured compounds with the metal–metal distances in corresponding pure metals at the same pressures (Extended Data Fig. 6) indeed shows a clear similarity. (In some cases—for example, in experimentally studied Fe_7_C_3_ at 158 GPa (ref. ^31^), or theoretically predicted Fe_7_N_3_ at 150 GPa (ref. ^32^)—the *A*–*A* distances are even slightly shorter in compounds than in pure metals). Notably, the average Re–N distance in NRe_6_ prisms in Re_7_N_3_ (⟨Re–N⟩ is 1.84 Å) follows the same trend as for other Th_7_Fe_3_-type structured compounds when ⟨*A*–*X* ⟩ is compared with ⟨*A*–*A*⟩ (Extended Data Fig. 6). According to our experimental data, the Re–N distances in trigonal prisms in Re_7_N_3_ vary from approximately 1.79 Å to 1.94 Å, as expected for pressures of several megabars (the shortest previously reported rhenium–nitrogen distance is approximately 1.96 Å in ReN_8_**·***x*N_2_ at 134 GPa)^11^. We note that in the TPa pressure range, the Re–Re interatomic distances become comparable with those of transition metals of the fourth period (Cr, Mn, Fe, Ni), which are known to form Th_7_Fe_3_-type structured (or similar) compounds at ambient (or relatively low) pressure^30^. It may be an indication that a huge reduction of the Re size promotes formation of Re_7_N_3_ at several hundreds of GPa, but the existence of Ru_7_B_3_ at ambient pressure^30^ (in ruthenium the metal–metal distance is approximately 2.68 Å versus approximately 2.75 Å in Re) suggests that the size factor may be important, but not necessarily crucial.

**Extended Data Fig. 7:**
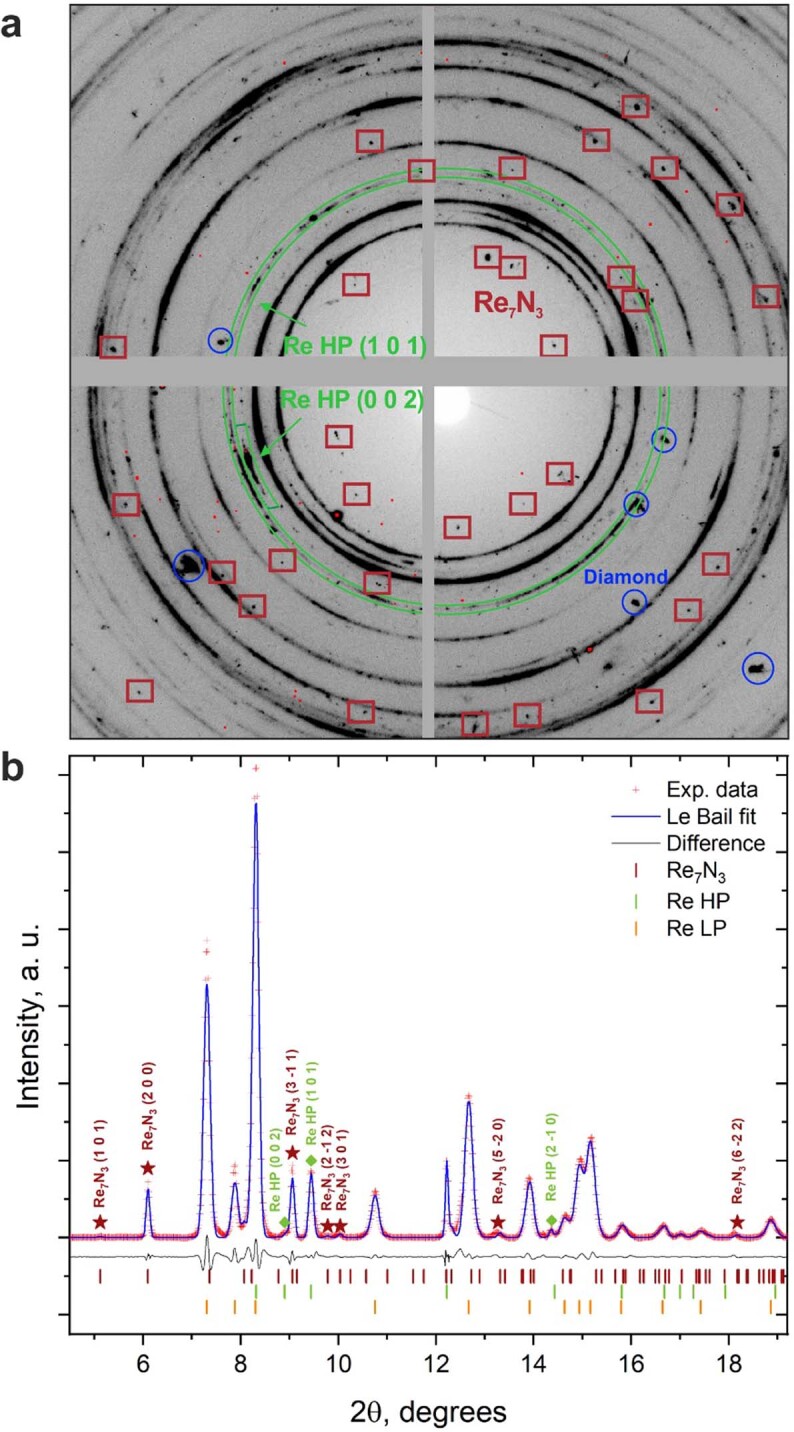
Example of powder diffraction pattern collected from dsDAC #2. Data were collected at ID11 (ESRF, Grenoble, France, beam FWHM 0.5 × 0.5 μm^2^) at 646 GPa (see Supplementary Table 1). **a**, 2D diffraction image shows diffraction spots of Re_7_N_3_ and rings of hexagonal close-packed (hcp) Re at different pressures. **b**, Refinement of powder diffraction pattern with Le Bail fit implemented in JANA2006 software. The values of the lattice parameters are given in Supplementary Table 3.

**Extended Data Fig. 8:**
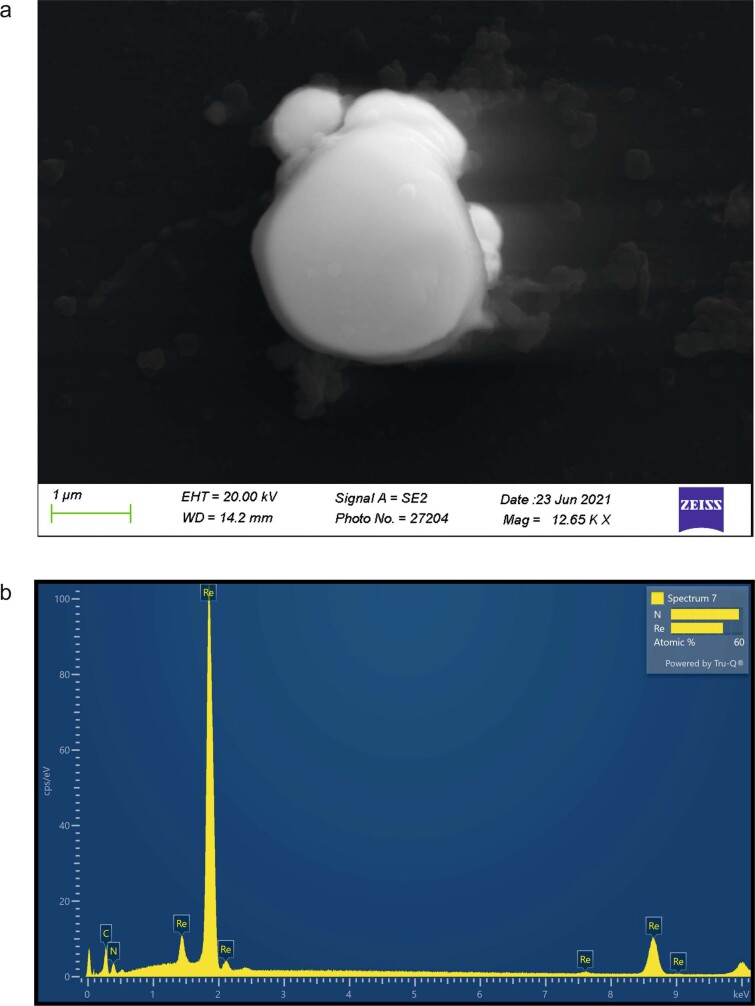
Characteristics of a sample extracted from the dsDAC #2. Sample contains Re and N in atomic proportions ~2:1. **a**, SEM image; **b**, example of EDX spectra. Images and spectra were collected on a ZEISS SEM, Leo Gemini 1530 with a Schottky field emission gun employing an accelerating voltage of 20 kV.

The synthesis of Re_7_N_3_ was reproduced in dsDAC #2. Diffraction data collected at ID11 at ESRF shows numerous diffraction spots, and the analysis of the integrated powder diffraction pattern confirmed the presence of the hexagonal phase with the lattice parameters very close to those obtained for Re_7_N_3_ in dsDAC #1 (Supplementary Tables 1, 3 and Extended Data Fig. 7). Unfortunately, the quality of the diffraction was insufficient for the single-crystal data analysis; the deterioration of the quality of diffraction data may be due to a pressure drop from around 140 GPa to 100 GPa on primary anvils upon laser-heating. Still, for dsDAC #2 we were able to release pressure to ambient without total destruction of the pressure chamber and found there a particle of almost 2 μm in diameter, which consisted of Re and N in the atomic ratio of about 2:1 (Extended Data Fig. 8). This finding provides additional evidence of the synthesis of rhenium nitride in dsDAC #2.

To elucidate the effect of the extreme compression on the stability of the Re_7_N_3_ compound and to characterize its physical properties, we carried out electronic structure calculations in the framework of density functional theory and studied its electronic, thermodynamic and vibrational properties (see Methods and Supplementary Information section ‘Computational details’). The optimized lattice parameters and coordinates of atoms of Re_7_N_3_ were found to be in excellent agreement with experiment (Supplementary Table 4). A difference in pressure calculated at experimental volumes for Re_7_N_3_ may indicate that the calculated equation of state of Re and/or Re_7_N_3_ at ultrahigh compressions is becoming less accurate, which is often the case in generalized gradient approximation calculations. Examination of the electronic band structure (Supplementary Information section ‘Electronic properties of Re_7_N_3_’ and Supplementary Fig. 1), electronic density of states (Supplementary Figs. 2, 3), electron localization function (Supplementary Fig. 4), and charge density maps (Supplementary Fig. 5) show that Re_7_N_3_ is a metal that has a combination of metallic and ionic bonding with some covalent component.

The dsDAC #3 was laser-heated to a maximum temperature of 3,450(200) K and the lattice parameters of Re measured after heating were found to be *a* = 2.2803(3) Å, *c* = 3.622(1) Å and *V* = 16.31(2) Å^3^. According to the equation of state^27^ of Re, the sample was under pressure of 730(4) GPa (Supplementary Table 1 and Supplementary Fig. 6). The analysis of single-crystal XRD data revealed the presence of a cubic phase (space group ${Fm}\bar{3}m$) with a lattice parameter of approximately 3.40 Å to approximately 3.41 Å depending on the spot from which the XRD pattern was taken. Structural solution suggests that the phase has an NaCl (B1)-type structure (Fig. 2 and Supplementary Fig. 7) with one position occupied by Re and the other by a light element. Attempts to refine the crystal structure assuming that the position of the light element is fully occupied by N or C led to an unreasonably high thermal parameter (approximately 0.1 Å^2^). For the highly symmetric NaCl-type structure containing heavy Re atoms, simultaneous refinement of the occupancy and the thermal parameter of a lighter element is not reasonable, so we constrained the thermal parameters of all atoms to be equal. In this approximation, the composition of the cubic phase was ReN_0.20_ (Supplementary Table 2). Of course, on the basis of XRD data alone we could not exclude that the light element might be carbon, but theoretical calculations (see Supplementary Information section ‘Re-based solution phase’) suggest that nitrogen is more plausible. A partial occupation of octahedral voids of the underlying face-centred cubic (fcc) packing of Re atoms by nitrogen predicts negative formation enthalpies of metastable alloys (Supplementary Figs. 8, 9 and Supplementary Table 5), whereas filling them with carbon leads to positive formation enthalpies (Supplementary Fig. 8 and Supplementary Table 6).

**Fig. 3:**
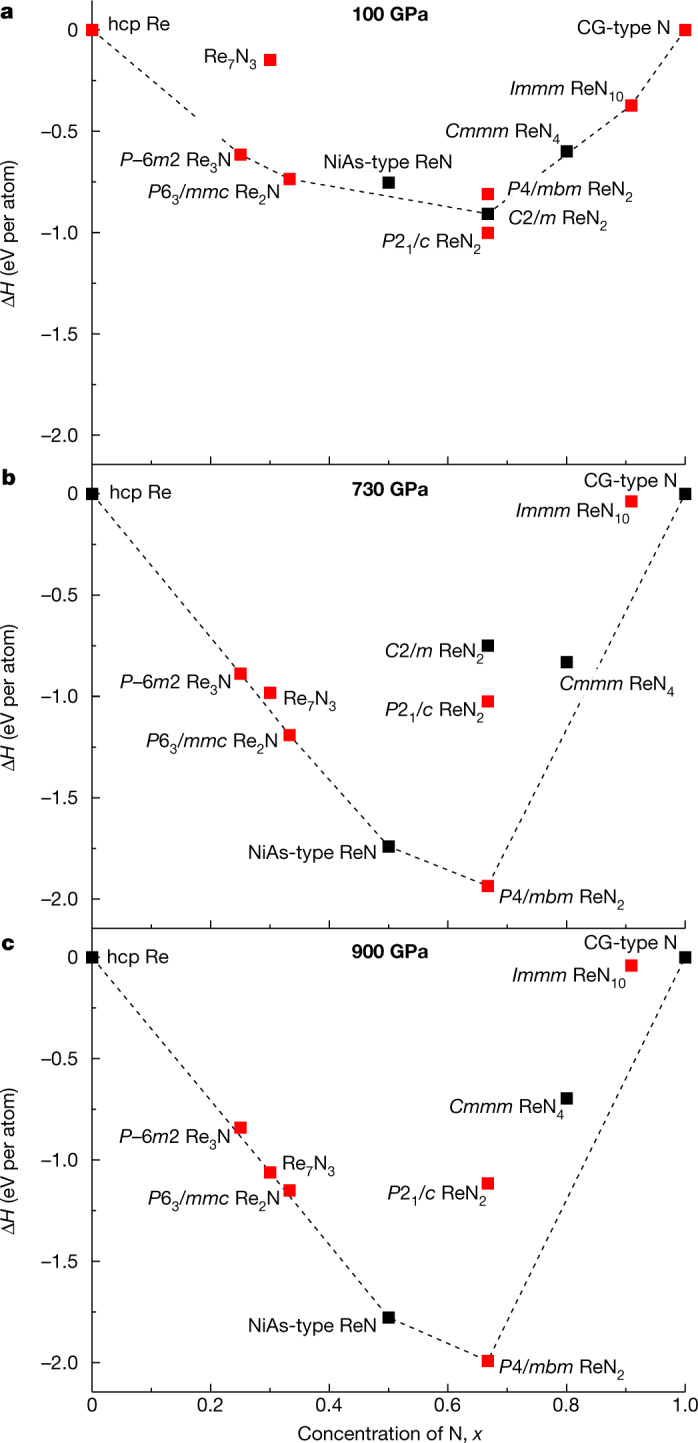
Formation enthalpy of Re_7_N_3_. **a**–**c**, Data are shown with respect to theoretically predicted^34^ (black squares) and experimentally known (red squares, Re_3_N and ReN_2_^13^ (*P*2_1_/*c*), ReN_2_ (*P*4/*mbm*), ReN_10_^11^ (*Immm*)) competing high-pressure phases in the ReN_*x*_ system, calculated at pressures of 100 GPa (**a**), 730 GPa (**b**) and 900 GPa (**c**). hcp, hexagonal close-packed; CG-type N, cubic gauche nitrogen.

**Extended Data Fig. 9:**
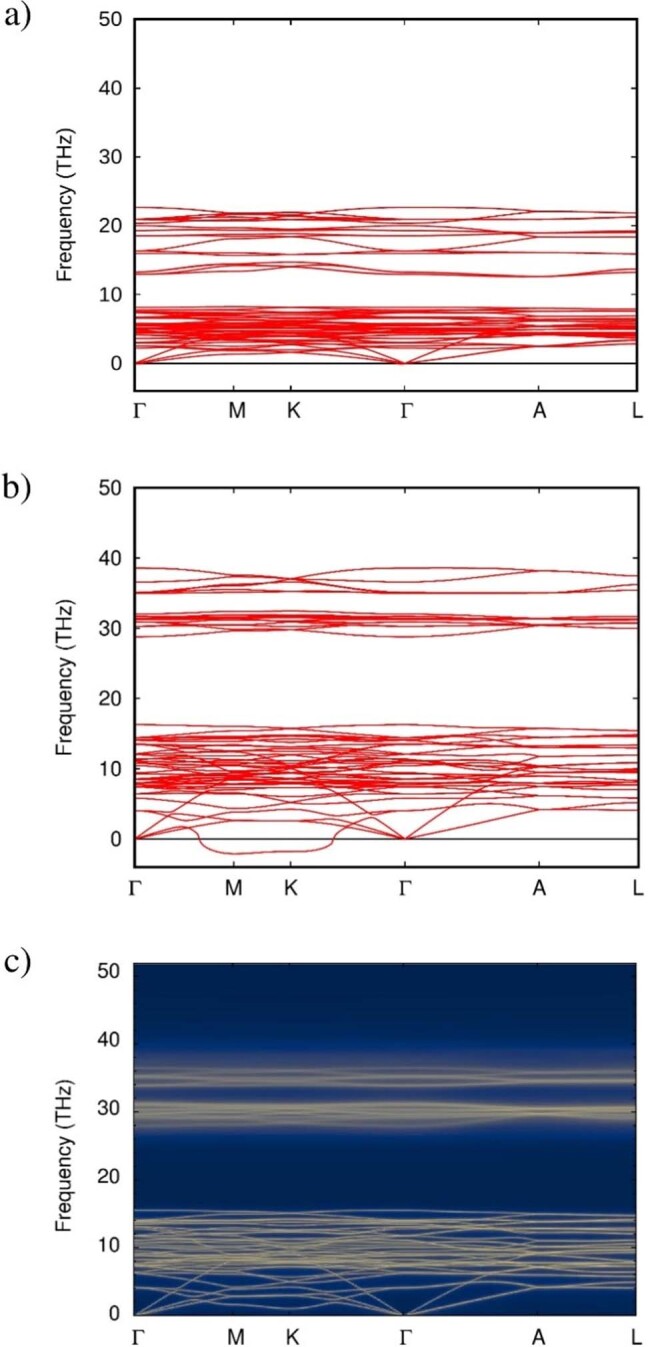
Phonon dispersion relations for Re_7_N_3_ calculated within the harmonic approximation. **a**, Theoretical pressure *P* = 102 GPa. **b**, Experimental volume 136.52 Å^3^, which corresponds to theoretical pressure ~730 GPa. **c**, Vibrational spectral function at *P* ≈ 730 GPa and *T* = 300 K, calculated using the temperature-dependent effective potential (TDEP) method. The results show that in the harmonic approximation, Re_7_N_3_ is unstable at the synthesis pressure (imaginary frequencies are shown below the zero-frequency line). Including anharmonic effects of lattice vibrations removes the dynamical instability at *P* = 730 GPa (all the branches are real).

Theoretical simulations enabled an insight into the possibility of synthesizing Re_7_N_3_ at pressures lower than those achieved in the current study. At 100 GPa the formation enthalpy of metastable Re_7_N_3_ is well above the convex hull (Fig. 3, Supplementary Information section ‘Thermodynamic stability of Re_7_N_3_’ and Extended Data Fig. 9). Even taking into account the anomalously large (approximately 0.2 eV per atom) metastability range of nitrides^33^, this compound cannot be considered as synthesizable at 100 GPa. By contrast, at 730 GPa the calculated formation enthalpy of Re_7_N_3_, although still above the convex hull, becomes well within the metastability range of nitrides (Fig 3, Supplementary Information section ‘Lattice dynamics of Re_7_N_3_’ and Extended Data Fig. 9), and at approximately 900 GPa—the pressure of the realized experimental synthesis—it lies on the convex hull (Fig. 3).

Pressures of more than several megabars have long been thought to have a profound effect on the chemistry and physics of materials^1,2^ and to lead to formation of phases with exotic crystal structures. In this work we have demonstrated that at pressures as high as those exceeding 600 GPa new compounds can be synthesized in laser-heated dsDACs and their structures can be solved in situ. By extending the experimental field of high-pressure synthesis and structural studies to the terapascal range, our work paves the way towards the discovery of new materials and observations of novel physical phenomena.

## Methods

Diffraction data were acquired at ID11 beamline at ESRF. Experiments with dsDAC #1 were performed using a Frelon 4M detector, wavelength 0.3099 Å, beam size 0.45 × 0.45 μm^2^ at FWHM; data for dsDAC #2 and dsDAC #3 were collected with Eiger2 CdTe 4M detector, wavelength 0.2882 Å, beam size 0.5 × 0.5 μm^2^ at FWHM. 2D mappings of still XRD images (without *ω* oscillations) were performed with an exposure time up to 10 s; single-crystal datasets were collected via DACs rotation around the *ω* axis from −38° to 38° with an angular step of 0.5° and an acquisition time of 10 s per step.

For the powder diffraction studies, calibration of instrument model and integration of diffraction patterns were made in the DIOPTAS^35^ software using CeO_2_ powder standard (NIST SRM 674b). Integrated patterns from powder XRD experiments were processed using the Le Bail technique implemented in JANA2006^36^ software. XRD imaging of the sample chamber was reconstructed using XDI^37^ programme and map of still images converted from ‘edf’ to ‘tif’ format. For the single-crystal XRD, integration of the reflection intensities and absorption corrections were performed in CrysAlisPro software^38^. A single crystal of orthoenstatite (Mg_1.93_,Fe_0.06_)(Si_1.93_,Al_0.06_)O_6_ (space group *Pbca*, *a* = 8.8117(2) Å, *b* = 5.18320(10) Å, *c* = 18.2391(3) Å) was used as calibration standard for refinement of the instrument model of the diffractometer. Diffraction images were converted from ‘edf’ to the native CrysAlisPro format ‘ESPERANTO’ with Freac software^38^. Detailed information of integration parameters as well as of the data-reduction output files and indicators of the XRD data quality are given in ref. ^19^. The crystal structures were solved using SHELXT or the superflip method in JANA2006 and Olex2^36,39,40^. Crystal structures were refined by least-squares minimization of adjustable parameters. We performed isotropic refinement of atomic displacement parameters due to limited dataset collected in DAC. Reflections coming from parasite diffraction produced by diamonds and crystallized pressure media were eliminated during the refinement procedure. The software Diamond^41^ was used for visualization of molecular graphics.

The electronic structure, total energy and forces calculations of the studied rhenium nitrides were carried out in the framework of density functional theory (see Supplementary Information section ‘Computational details’). We used supercells of different sizes with an underlying fcc crystal structure and various amounts of either N or C to simulate the Re–N and Re–C cubic phases with NaCl (B1)-type structure (see Supplementary Information section ‘Re-based solution phase’). To investigate the influence of pressure on the thermodynamic stability of Re_7_N_3_, its enthalpy of formation, as well as the enthalpies of formation for various phases of rhenium nitride, known experimentally^10,11,13^ and predicted theoretically^34^, were calculated and a thermodynamic convex hull was constructed based on the calculation results (Supplementary Information section ‘Thermodynamic stability of Re_7_N_3_’).

Phonon dispersion relations for Re_7_N_3_ were calculated in the harmonic approximation at volume 200 Å^3^ (*a* = 7.122 Å, *c* = 4.553 Å) of the unit cell, corresponding to *P* = 102 GPa, as well as at experimental volume 136.52 Å^3^ (*a* = 6.277 Å, *c* = 4.001 Å) of the unit cell (Supplementary Table 4), which corresponded to calculated pressure 732 GPa (see Extended Data Fig. 9 and Supplementary Information section ‘Computational details’). Because Re_7_N_3_ is predicted to be dynamically unstable at the synthesis pressure owing to the presence of imaginary frequencies in this approximation (Extended Data Fig. 9 and Supplementary Information section ‘Lattice dynamics of Re_7_N_3_’), we investigated the anharmonic effects of lattice vibrations at finite temperature using the temperature-dependent effective potential (TDEP) method^42^ with effective second-order and third-order interatomic force constants calculated from first principles^43^. The calculations are based on modelling the potential energy surface in the vicinity of equilibrium with a Hamiltonian of the form:

1 $$H={U}_{0}+\sum _{i}\frac{{{\bf{p}}}_{i}^{2}}{2{m}_{i}}+\frac{1}{2!}\sum _{ij\alpha \beta }{\varPhi }_{ij}^{\alpha \beta }{u}_{i}^{\alpha }{u}_{j}^{\beta }+\frac{1}{3!}\sum _{ijk\alpha \beta \gamma }{\varPhi }_{ijk}^{\alpha \beta \gamma }{u}_{i}^{\alpha }{u}_{j}^{\beta }{u}_{k}^{\gamma }+\ldots ,$$

where **p** and *m* are the momentum and the mass of ion *i*, respectively, *Ф* are interaction parameters (the effective force constants) ofincreasing order, *u* denotes the displacement of ions (*i*, *j* or *k*) from their equilibrium positions, and *αβγ* are Cartesian components.

We calculated the spectral function *S*(**q**, *E*) at 300 K, which describes the spectrum of lattice excitations with energy *E* = *ħΩ* (*Ω* is the applied frequency) for mode *s* with harmonic frequency *ω*_**q***s*_ at wavevector **q** (refs. ^44,45^). *S*(**q**, *E*) provides insight into the phonon frequencies as well as strength of three-phonon processes via the broadening in Extended Data Fig. 9. The *S*(**q**, *E*) of Re_7_N_3_ is typical of a weakly anharmonic solid with Lorentzian broadening of single peaks. Additionally, the lines are reasonably crisp, without substantial broadening, indicating that the anharmonic interaction strength is well within the range of validity for the perturbation theory. Importantly, Re_7_N_3_ is seen to be dynamically stable (there are no imaginary frequencies) at the synthesis pressure (see Supplementary Information section ‘Lattice dynamics of Re_7_N_3_’).

### Reporting summary

Further information on research design is available in the Nature Research Reporting Summary linked to this paper.

## Online content

Any methods, additional references, Nature Research reporting summaries, source data, extended data, supplementary information, acknowledgements, peer review information; details of author contributions and competing interests; and statements of data and code availability are available at 10.1038/s41586-022-04550-2.

## Supplementary information

Supplementary Information Supplementary Information contains sections describing ‘Computational details’, ‘Electronic properties of Re_7_N_3_’, ‘Re-based solution phase’, ‘Thermodynamic stability of Re_7_N_3_’, ‘Lattice dynamics of Re_7_N_3_’ and ‘Brief overview of the double-stage DAC (dsDAC) technique’; Supplementary Figs. 1–9, Tables 1–7 and references.

Reporting Summary

## Data Availability

Data supporting this work are available at Zenodo, 10.5281/zenodo.5899162. Structural data deposit at Cambridge Crystallographic Data Centre (CCDC), CSD-2143754 (10.25505/fiz.icsd.cc29yrcd).

## Code availability

The temperature-dependent effective potential method is implemented as a package that deals with finite-temperature lattice dynamics in solids. The package is released under the MIT license, available on GitHub, see https://ollehellman.github.io. Source files for TDEP 1.1 are located at https://ollehellman.github.io/lists/files.html.
