# Supplementary material for: Materials synthesis at terapascal static pressures
Source: Nature. 2022 May 11;605(7909):274–8. doi: 10.1038/s41586-022-04550-2 (PMC9095484; doi:10.1038/s41586-022-04550-2)
Supplement: Supplementary file 1 — Supplementary Information contains sections describing ‘Computational details’, ‘Electronic properties of Re7N3’, ‘Re-based solution phase’, ‘Thermodynamic stability of Re7N3’, ‘Lattice dynamics of Re7N3’ and ‘Brief overview of the double-stage DAC (dsDAC) technique’; Supplementary Figs. 1–9, Tables 1–7 and references. [file 41586_2022_4550_MOESM1_ESM.pdf]

nature portfolio

https://doi.org/10.1038/s41586-022-04550-2

Supplementary information

Materials synthesis at terapascal static pressures

In the format provided by the
authors and unedited

Supplementary Information

Computational details

We employed the projector augmented wave (PAW) method[1,2] as implemented in the VASP[3]. The exchange and correlation effects were considered using generalized gradient approximation (GGA) with PBE parametrization[4]. To investigate the high-pressure phases, we used "hard" potentials with the configurations of the valence electrons of $5p^6 5d^6 6s^1$ and $2s^2 2p^6$ (Re_pv and N_h potentials) for rhenium and nitrogen, respectively. The cut-off energy was set to 870 eV. The integration over the Brillouin zone was performed using the k-points obtained by Monkhorst-Pack method[5] with the 33×33×33 points mesh for Re_7N_3, 30×30×20 for *hcp*-Re, 28×28×12 for $P\bar{6}m2$ Re_3N, 30×30×10 for *P6₃/mmc* Re_2N, 5×10×5 for $Re_{16}N_4$, 30×30×10 for $P\bar{6}m2$ Re_3N_2, 32×32×16 for NiAs-type ReN, 8×20×10 for *C/2m* ReN_2, 12×8×12 for $P2_1/c$ ReN_2, 10×19×11 for *Imm2* ReN_3, 12×24×36 for *Cmmm* ReN_4, 14×8×14 for *Immm* ReN_{10}, 20×20×20 for diamond C, 16×16×16 for *cg*–type N. All initial configurations of the compressed crystals were optimized with respect to lattice parameters and atomic coordinates to ensure the hydrostatic pressure condition. Methfessel-Paxton algorithm[6] with broadening 0.1 eV was used for the structure optimization, electronic band structure and the enthalpies calculations. The tetrahedron method for the Brillouin zone integration with Blöchl corrections[7] was applied for calculations of the electronic density of states.

Harmonic phonons for Re_7N_3 were calculated using the small displacement method implemented into Phonopy software package[8,9]. Phonon dispersions have been calculated on a 2×2×2 supercell and 3×3×3 sampling of the Brillouin zone. Methfessel-Paxton algorithm[6] with broadening 0.2 eV was used for the force field calculation. Additional calculations carried out with a (2×2×3) supercell size and (3×3×3) sampling of the Brillouin zone (not shown) confirm sufficient convergence of the results obtained on the smaller supercell, which are presented in Extended Data Fig. 9.

To investigate the anharmonic effects of lattice vibrations at finite temperature we employed the Temperature Dependent Effective Potential (TDEP) method[11-13]. A calculation of force constants (see Methods, Eq. (1)) consists of creating snapshots of a supercell with thermally displaced atoms corresponding to a specified temperature, calculating forces using an *ab initio*

density functional theory code such as VASP, and fitting the forces and displacements with the model Hamiltonian. Performing this procedure at given temperature renormalizes the interaction parameters, incorporating all orders of non-harmonic effects. Once force constants are obtained, we can obtain the phonon spectra, broadening and linewidth needed for calculation of the spectral function S(**q**,E).

Re_7N_3 structure is modeled by supercell containing 260 atoms in total, with 182 Re atoms and 78 N atoms. For given experimental volume 136.52 $Å^3$ (calculated pressure 732 GPa), we performed the iterative scheme to obtain a set of forces and atomic displacements for calculation of the second and the third order force constants using TDEP. An efficient stochastic sampling approach to prepare a set of thermalized with thermal displacements corresponding to Maxwell-Boltzmann statistics at temperature 300 K configurations for each iteration. Once the snapshots are created, we performed a series of first-principles simulations to obtain a set of force-displacement data sets.

We performed iterative scheme which consists of 6 iterations with 200 snapshots each. Note that the plane wave energy cutoff used in our VASP calculations was set to 600 eV and the Brillouin zone was sampled with a 3×3×3 k-point mesh. Using the output from the final iteration we calculated the spectral function at T=300 K shown in Extended Data Fig. 9.

Electronic properties of Re_7N_3

The electronic band structure of Re_7N_3 is shown in Supplementary Information Fig. S1, while its electronic density of states (DOS) is shown in Supplementary Information Fig. S2 and compared with that of pure *hcp*-Re in Fig. S3. The nitride is metallic owing to the bands crossing the Fermi energy E_F (Supplementary Information Fig. S1) leading to the finite DOS at E_F (Supplementary Information Fig. S2). The main contribution to the DOS at the Fermi level comes from 5*d* electrons of Re. The peaks in the lower energy part of the DOS (-24 eV: -20 eV) arise from 2*s* electrons of N (Supplementary Information Fig. S3). This band is separated by an energy gap of 4.5 eV from states (-16 eV: -11 eV) formed predominantly by N *p*-electrons and Re d-electrons, with some contribution from Re *s*- and *p*-states. Note that Re states could be partially related to the N states decomposed into Re-orbitals (Fig. S3). A broad band (-11 eV: 9 eV) is predominantly due to Re *d*-electros.

Due to the difference in electronegativity between Re and N, one can expect that the bonding in the system should be predominantly ionic. On the other hand, the overlap between *d*-orbitals of Re and *p*-orbitals of N can be considered as an indicator of hybridization and the covalent component of the bonding. Indeed, the presence of unoccupied N *p*-states above the Fermi energy shows that the interaction cannot be purely ionic. To better understand the nature of atomic bonding in Re_7N_3, we calculated the electron localization function (ELF) (Supplementary Information Fig. S4), spatial distribution of electronic density maps and total charge-density contour (Fig. S5). The low localization of electrons in the region between adjacent N and Re atoms with almost spherically distributed ELF attractors around nitrogen atoms indicates the ionic component of the interatomic bond (Supplementary Information Fig. S4). The same features can be seen from Supplementary Information Fig. S5, which shows the spatial distribution of electronic density in the range [−16 eV: -10 eV], namely high and nearly spherical charge distribution around N atoms. Next, in the energy range [-10 eV: -4 eV] there are predominantly strong Re-Re and weaker N-N interactions (Supplementary Information Fig. 5). In the interval [-4 eV: 0 eV] an increase in electron density between Re and N atoms and directional charge redistribution are observed (Supplementary Information Fig. S5), which indicates the presence of a covalent component of the atomic bond. Finally, the total charge density plot (Supplementary Information Fig. S5) shows slightly anisotropic spherically distributed areas of charge density around Re and N atoms with bridges between them.

Re-based solution phase

We use VASP to calculate the mixing enthalpies for 2×1×2 supercells with an underlying *fcc* crystal structure with 16 Re atoms and various amounts of either N or C ($Re_{16}(C,N)_x$ with x=2, 3, 4) occupying octapores in the supercells to simulate the Re-N and Re-C cubic phases with NaCl (B1) type structure. To calculate the formation enthalpy ΔH for Re-N solid solutions, we used *hcp*-rhenium and nitrogen in the cg-N structure and for Re-C carbon in the diamond structure.

Analysis of the 2×1×2 *fcc* configurations containing nitrogen shows that it is beneficial for it to be either in neighboring octopores (touching along the edge), or in the octopores that are on the third coordination sphere (that is, those which don't touch at all, but are not far away). Such configurations lead to negative formation enthalpies (Supplementary Information Fig. S8,

Supplementary Information Table S5). The situation when the nitrogen octopores touch only in a single vertex (the case, where each unit cell stoichiometry is Re_4N) is the least energetically favorable.

We note that for each individual supercell structural relaxation leads to slight orthorhombic distortions (< 4%). However, the presence of several nearly energetically degenerate supercells (Supplementary Information Table S5) suggests that the distortions could be out in a macroscopic sample. Therefore, we perform additional structural relaxations in 2×2×2 supercells using Quantum Espresso[14]. Similar to the VASP calculations , we use the projector augmented wave (PAW) method and consider exchange and correlation effects in terms of the generalized gradient approximation (GGA) with PBE parametrization[4] . We use a kinetic energy cutoff of 80 Ry for wavefunctions and 800 Ry for the charge density and 2×2×2 *k*-points.

We generate all possible structures based on a 2×2×2 supercell of *fcc*-Re with stoichiometry $Re_{32}N_8$, which showed the most negative formation enthalpies of the probed 2×1×2 supercells (Supplementary Information Fig. S8, Supplementary Information Table S5). When occupying 8 out of 32 possible octopores in the cell, it is possible to build 10518300 structures. We reduce the number by considering only structures in which the center of gravity of the nitrogen coordinates coincides with the center of the supercell, which reduces the data set to ~50000 structures and ensures a relatively uniform distribution of N atoms (Supplementary Information Fig. S9). From this set, we randomly choose 100 structures and perform a structural relaxation with no constraints on the lattice or cell parameters until forces are < 0.5 meV/Å^2 per atom. Within the data set, we obtain a small enthalpy variation, depending on the relative position of the octopores occupied by N, in agreement with the 2×1×2unit cell calculations. All calculations maintain the *fcc*-Re host lattice with varying orthorhombic distortion between 0.4% and 4% and < 0.3% variation in cell volume. Averaging over all coordinates leads to a nearly cubic structure (average orthorhombic distortion is below 0.02%).

Thus, the formation of $ReN_{0.20}$ solution phase with NaCl (B1) type structure is quite plausible, even though the alloy should be metastable at the synthesis pressure as its mixing enthalpy is above the convex hull. For all ReC solid solutions, the formation enthalpies are positive, making the formation of the Re-C alloys in the experiment highly unlikely (Supplementary Information Fig. 8, Supplementary Information Table S6).

Thermodynamic stability of Re_7N_3

Investigation of the influence of pressure on the thermodynamic stability of Re_7N_3 is a highly non-trivial task. In the maximum pressure range 100 GPa-200 GPa that has been achieved so far, nitrides of rhenium can be found in a wide range of compositions as a consequence of the multiple oxidation states of rhenium, while the phase diagram in the TPa pressure range is unknown and an identification of all the competing phases, as well as a treatment of the off-stoichiometric phases is beyond the subject matter of the present study. Therefore, the enthalpies of formation were calculated for Re_7N_3 and compared to experimentally known and theoretically predicted stoichiometric high-pressure phases in Re-N system from the literature. In more details, we used hcp rhenium in the *P6₃/mmc* structure, and nitrogen in the *cg*-N structure. Zhao et al.[15] previously constructed a convex hull at a pressure of 100 GPa[16]. Using the evolutionary structure search method in a region with a high rhenium content, two thermodynamically stable structures, Re_3N ($P\bar{6}m2$) and Re_2N ($P6_3/mmc$), were identified by Zhao *et al.* theoretically. In fact, these two compounds were synthesized earlier by Friedrich *et al.*[10] at lower pressures (13-31 GPa). Two nitrides Re_3N_2 ($P\bar{6}m2$) and ReN-NiAs were found in ref. [15] to be quite close to the convex hull, both slightly above it. Thus, these four structures together with Re_7N_3 were considered in the present study.

In the region enriched with nitrogen we considered the following phases: ReN_2 (*C2/m*), ReN_3 (*Imm2*), ReN_4 (*Cmmm*), ReN_2 ($P2_1/c$), ReN_2 (*P4/mbm*), ReN_{10} (*Immm*). The three former phases, ReN_2 (*C2/m*), ReN_3 (*Imm2*) and ReN_4 (*Cmmm*), were predicted theoretically as thermodynamically stable phases at 100 GPa in ref. [15]. The three latter compounds, ReN_2[17] (*P21/c*), ReN_2 (*P4/mbm*), and ReN_{10}[18] (*Immm*), have been recently synthesized experimentally. Our results, summarized in Fig 3 show that an increase in pressure leads to remarkable increase of stability of Re_7N_3 with respect to the other competing phases.

The results of our calculations at a pressure of 730 GPa (corresponding to calculated pressure at the experimental synthesis volume) are shown in Fig. 3b. Two nitrides, Re_3N and Re_2N, lie on a convex hull. The enthalpy of formation of Re_7N_3 lies slightly above the ground state line (~ 0.05 eV) between Re_3N and Re_2N. The compound Re_3N_2 ($P\bar{6}m2$) lies far from the ground state

line, while ReN in the NiAs-type structure becomes thermodynamically stable at this pressure (among the considered phases). In a region with a high nitrogen content, the convex hull line passes only through ReN_2 nitride (*P4/mbm*).

Since the enthalpy of formation for Re_7N_3 is very close to the ground state line, a change in pressure can affect the thermodynamic stability of this nitride. To study this further, we constructed a ground state line at higher pressure of 900 GPa (Fig. 3). In the concentration interval enriched with nitrogen the results changed very little. However, for nitrides with a high rhenium content changes were significant. It can be seen that at this pressure the Re_2N and ReN phases are still thermodynamically stable, but Re_3N turned out to lie slightly higher than the ground state line. Most importantly, for Re_7N_3 the change in pressure placed its enthalpy of formation at the convex hull line. Note that at P=100 GPa Re_7N_3 has formation enthalpy wich is well above the convex hull line (Fig. 3).

Lattice dynamics of Re_7N_3

It is necessary that the Re_7N_3 phase is at least metastable to be synthesized in experiment. This can be evaluated theoretically in a study of the dynamical stability of a material: in a metastable phase all the frequencies of its lattice vibrations are real. To check the dynamical stability of the Re_7N_3, we calculated its phonon dispersion relations in the harmonic approximation. We observed that at 100 GPa the dynamical stability condition is fulfilled (Extended Data Fig. 9), despite the fact that the formation enthalpy of Re_7N_3 is well above the convex hull (Fig. 3). Surprisingly, calculations carried out at ~730 GPa, that corresponds to volume per atom at the experimental synthesis pressures, showed the presence of imaginary frequencies along Γ-M-K-Γ direction of the Brillouin zone (Extended Data Fig. 9). This means that in the harmonic approximation Re_7N_3 would be classified as dynamically unstable, impossible to synthesize compound. However, calculations of the vibrational spectral function carried out at T=300 K that take into account anharmonic effects of lattice vibrations do not show any sign of the dynamical instability and confirm that Re_7N_3 is at least metastable at this pressure (Extended Data Fig. 9).

This effect is interesting, giving the fact that the temperature 300 K corresponds to ~25 meV, while the *PV* term at 1 TPa is ~750 eV. Moreover, Re_7N_3 is not a strongly anharmonic solid:

phonon lifetimes are quite long, as seen from a relatively small broadening of the phonon lines in (Extended Data Fig. 9). The contribution of anharmonic effects of lattice vibrations is expected to be tiny in such materials. To understand this observation, we consider in more details the electronic structure of the Re_7N_3 and its interplay with lattice dynamics of this system. We recall that Re electron configuration is Xe $4f^{14}$ $5d^5$ $6s^2$. Nine electrons are transferred from seven Re atoms to three N atoms. The remaining sp- and d-electrons are weakly hybridized, leading to a nearly half-filled Re *d*-band, similar to Re metal. Indeed, comparing the Re *d*-band of Re_7N_3 with the DOS of pure *hcp*-Re calculated at nearly the same pressure (Supplementary Information Fig. S3). The latter, being half-filled by 5 Re *d*-electrons that occupy all the bonding states leaving all the anti-bonding states unoccupied explains very high formation energy of Re-metal in the framework of the Friedel rectangular band model[19]. Even more well-developed separation of the bonding states below the Fermi energy from antibonding states above the Fermi energy by a pseudogap located in a vicinity of E_F is seen in the DOS of Re_7N_3.

In fact, comparing its electronic DOS at the synthesis pressure with that calculated at P~100 GPa one observes (Supplementary Information Fig. 2), besides a typical pressure induced broadening of the bands, a clear shift of the occupied peaks down in energy (relative to E_F, see insets in Supplementary Information Fig. 2). Such a position of the pseudogap, in general, is a characteristic of a stable system, while the pressure-induced shift of the occupied peaks reduces the one-electron contribution to the total energy contributing to the pressure-induced stabilization of Re_7N_3.

However, as we deal with a chemically complex compound with many atoms per unit cell, the details of the electronic structure are non-trivial. We identify the presence of a Van-Hove singularity seen as a peak of the electronic DOS at the Fermi level (inset in Supplementary Information Fig. S2), associated with rather flat bands along Γ-M line (Supplementary Information Fig. S1). Though the singularity is small, its position at the Fermi energy is quite unfavorable from an energetic point of view, contributing to the observed dynamical instability of Re_7N_3 in the harmonic approximation at P~730 GPa (Extended Data Fig. 9) which effectively uses the static ideal crystal lattice (with small displacements of selected ions) for calculations of the force constants. Note that at P~100 GPa the Van-Hove singularity is above E_F, and Re_7N_3 is predicted to be dynamically stable in the harmonic approximation. On the other hand, it has been established

that lattice vibrations at finite temperature smear out the singularities of the electronic structure, leading to a disappearance of anomalies at the phonon dispersion relations[20]. As the Van-Hove singularity at the Fermi level observed at P~730 GPa is quite small in Re_7N_3, the TDEP calculations at temperature 300 K predict that the materials is dynamically stable at the synthesis pressure (Extended Data Fig. 9).

Brief overview of the double-stage DAC (dsDAC) technique

The ds-DAC original purpose was to generate ultra-high static pressures, those beyond the limit of a conventional DAC of about 300 to maximum 400 GPa. At first, pressures of about 600 GPa were achieved in a ds-DAC[21], as determined on the lattice parameters of gold using powder X-ray diffraction (XRD). Then static equations of states (EOSes) of several metals (platinum, osmium, tungsten, and tantalum) were cross-calibrated up to 500 GPa[22] based on the EOS of gold by Yokoo et al.[23] and the compressional behavior of Os was studied up to 774 GPa[22]. Hitherto, the highest static pressure achieved using ds-DACs is 1065 GPa[24], as determined on the gold pressure scale[23]. Modified ds-DAC designs tried by various research groups[25–27] have not led to generating pressures comparable to those reported by Bayreuth team[24]. Sakai et al.[28] conducted experiments in a ds-DAC with the secondary anvils manufactured from single crystal diamond using FIB and reported the maxim pressure of 460 GPa. Although these authors achieved the same degree of compression of rhenium as in Dubrovinsky et al.[21], they reported different pressures, as they used the Re pressure scale of Anzellini et al.[29]. There is a significant inconstancy between the data of Anzellini et al.[29] and Dubrovinsky et al.[21] and this reflects some general difficulties in the pressure characterization at multimegabar pressures. A unique estimate of pressure becomes difficult, if there are discrepancies in the literature. This is the case for Re, as the results by Sakai et al.[28] up to ~300 GPa pressure are close to those of Anzellini et al.[29], while Jenei's et al.[30] measurements in the same pressure range agree with the EOS of Dubrovinsky et al.[21].

Recently diamond anvils of a toroidal shape (t-DACs) were described by Jenei et al.[30] and Dewaele et al.[31] The both groups[30,31] were able to generate static pressures above 600 GPa, thus confirming conclusions of Bayreuth team that pressures above 0.5 TPa may be achieved using the DAC technique. As pointed out by Dewaele et al.[31], the toroidal shape of the diamond anvil of the t-DAC has similarities with the second-stage diamond anvil of the Bayreuth's ds-DAC[21,22,24].

Supplementary Information Figures

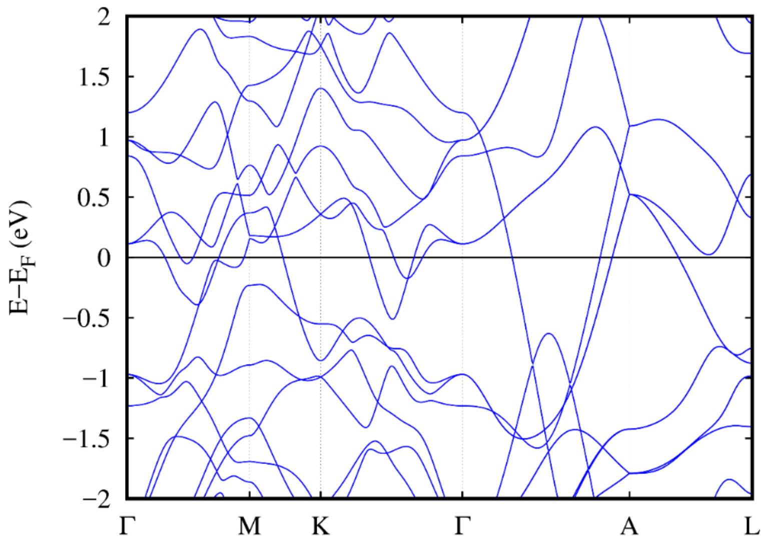

Supplementary Information Figure S1. Electronic band structure of Re_7N_3 calculated at experimental volume 136.5 Å^3 corresponding to calculated pressure ~730 GPa. E_F denotes the Fermi energy.

a)

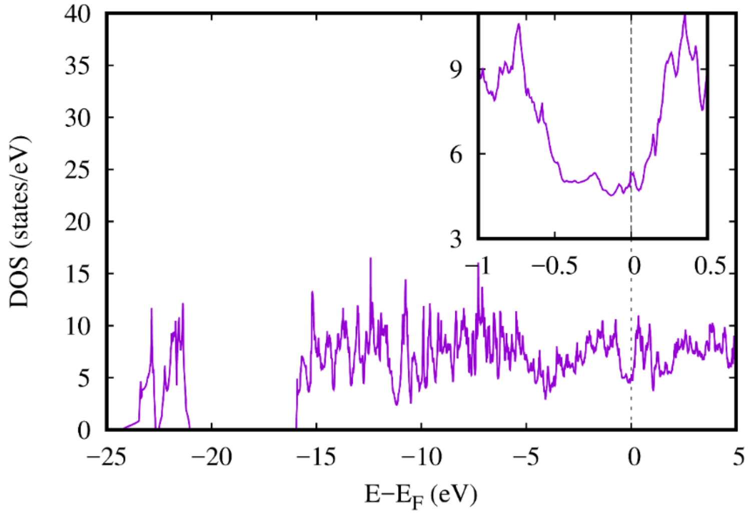

b)

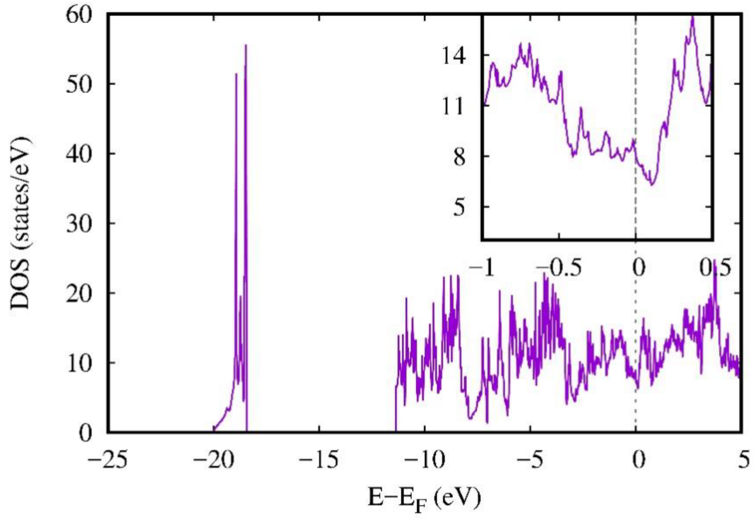

Supplementary Information Fig. S2. (a) Electronic density of states (DOS) of Re_7N_3 calculated per formula unit as a function of energy E at experimental volume 136.5 Å^3 corresponding to calculated pressure ~730 GPa. The inset shows magnified image of the eDOS in a vicinity of the Fermi energy E_F. (b) DOS of Re_7N_3 calculated per formula unit at volume 200 Å^3 corresponding to calculated pressure ~100 GPa is shown for comparison.

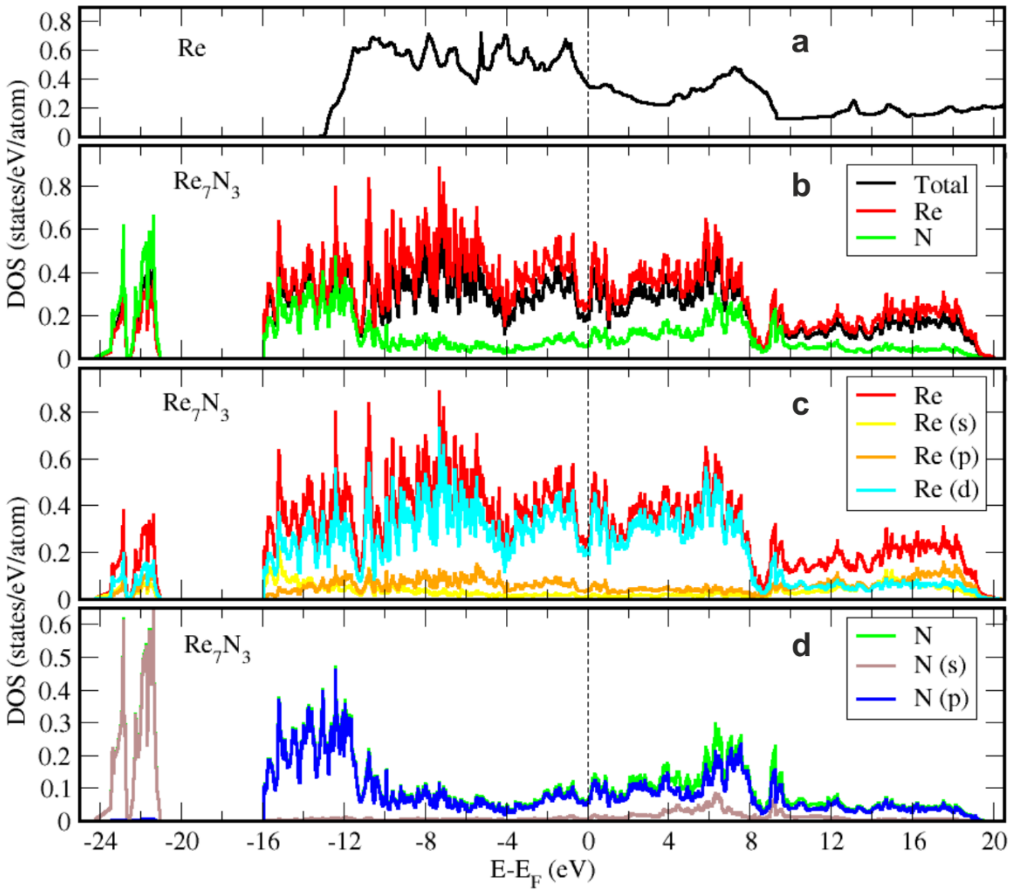

Supplementary Information Figure S3**.** Electronic density of states calculated as a function of energy E for hcp Re at V=16.95 Å^3 (P=734 GPa) (a) and local partial eDOS of Re_7N_3 at V=136.5 Å^3 (P=732 GPa) (b-d). (b) Total and local (atom-projected) densities of states of the Re_7N_3. (c) Partial (orbital-projected) local density of states at Re atoms. (d) Partial local density of states at N atoms. Energy zero is chosen at the Fermi energy E_F.

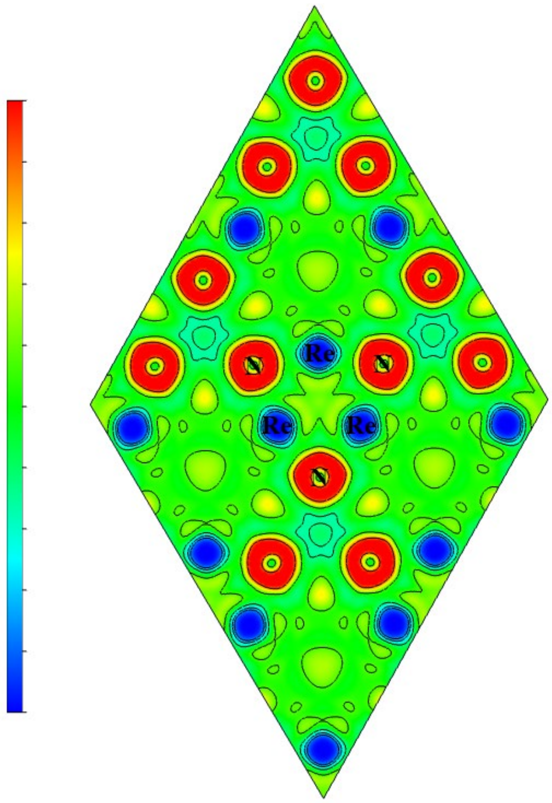

Supplementary Information Figure S4. Electron localization function of Re_7N_3 calculated at experimental synthesis volume 136.5 Å^3 corresponding to calculated pressure ~730 GPa. The saturation level value is 0.7.

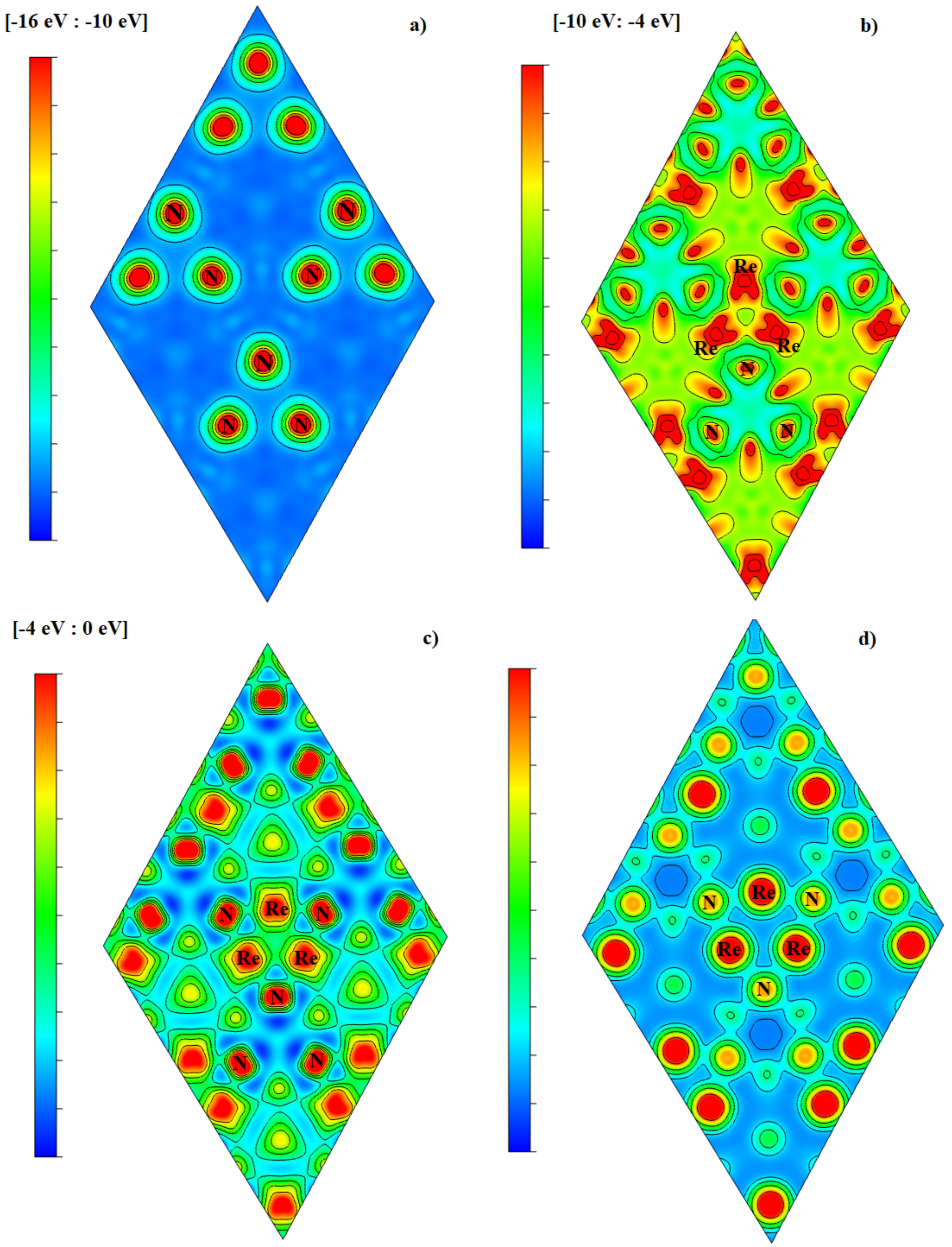

Supplementary Information Figure S5. Spatial distributions of charge density of Re_7N_3 in energy regions [−16 eV: -10 eV] (a), [−10 eV: -4 eV] (b), and [−4 eV: -0 eV] below the Fermi energy (c), as well as total electronic density map (d) calculated at experimental synthesis volume 136.5 Å^3 corresponding to calculated pressure ~730 GPa.

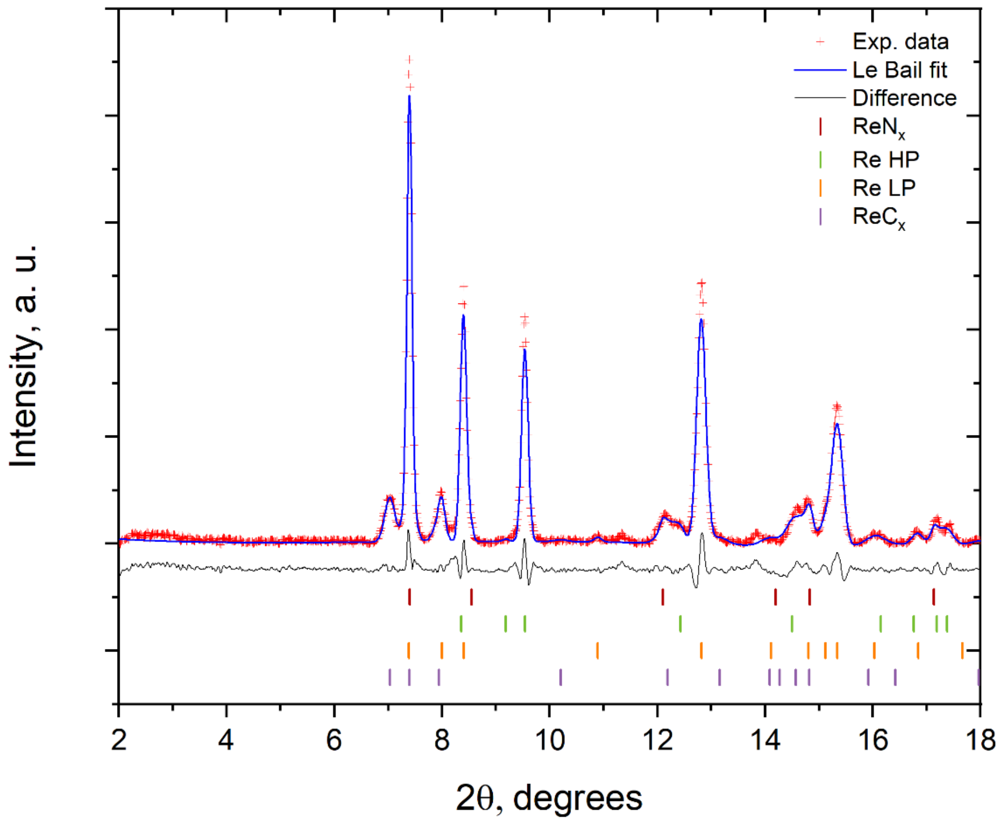

Supplementary Information Fig. S6. Example of a powder diffraction pattern collected from dsDAC #3 at ID11 (ESRF, Grenoble, France, the beam of 0.5x0.5 μm^2 FWHM and the wavelength 0.2952 Å). Refinement performed with Le Bail fit implemented in Jana2006 software. Values of lattice parameters are given in Supplementary Information Table S7. Weak and relatively broad reflections of hexagonal phase are assigned to ReC_x (x≈0.6) as far as similar phase was reported in ref.[32] as a product of chemical interaction of rhenium and carbon in the similar pressure range. Composition of the cubic ReN_x phase is approximately $ReN_{0.2}$.

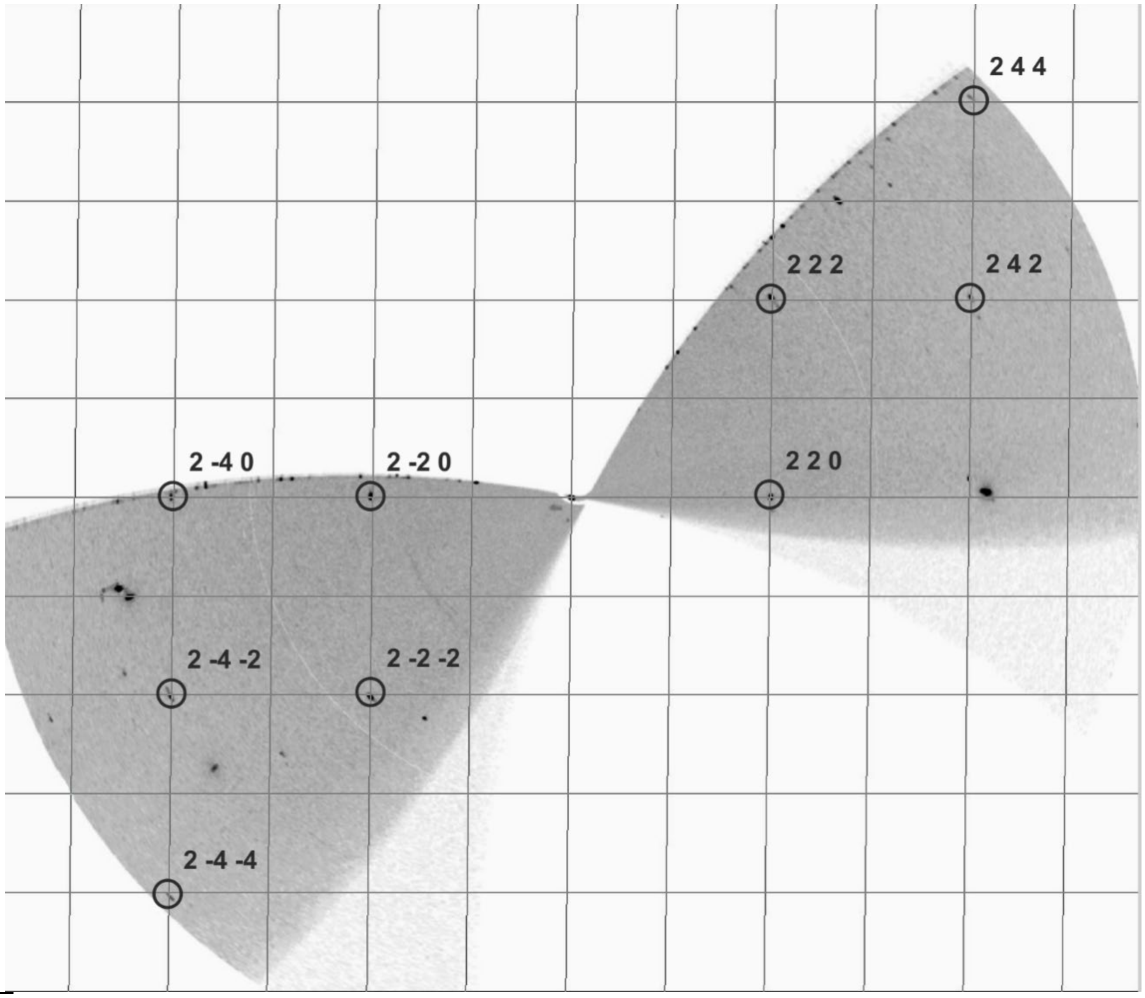

Supplementary Information Figure S7. Example of reciprocal space reconstructions (*2kl* plane) for the cubic phase (space group Fm$\bar{3}$m, lattice parameter a=3.3994(7) Å) found in dsDAC #3.

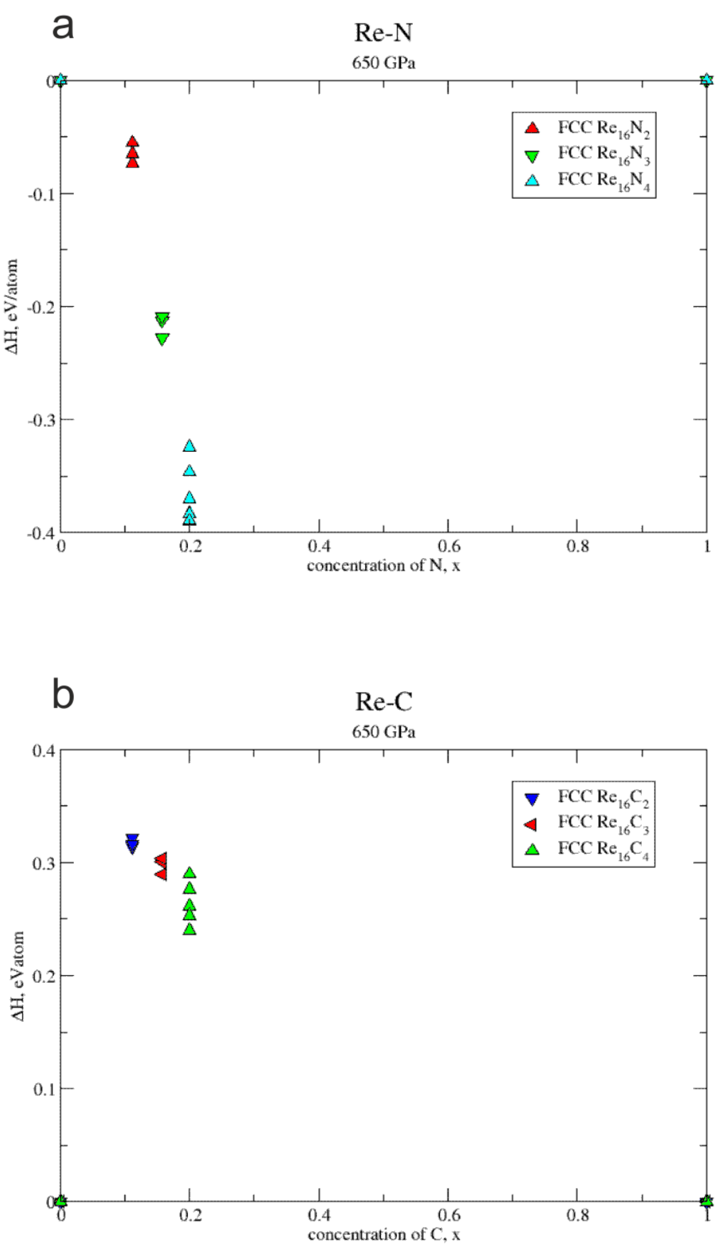

Supplementary Information Figure S8. Formation enthalpy for the most energetically favorable configurations of Re-N (a) and Re-C (b) 2×1×2 supercells simulating Re-N cubic phase with NaCl (B1) type structure. List of formation enthalpies for all 2×1×2 supercells considered in this study can be found in Supplementary Information Table S5 and Table S6 for N and C, respectively.

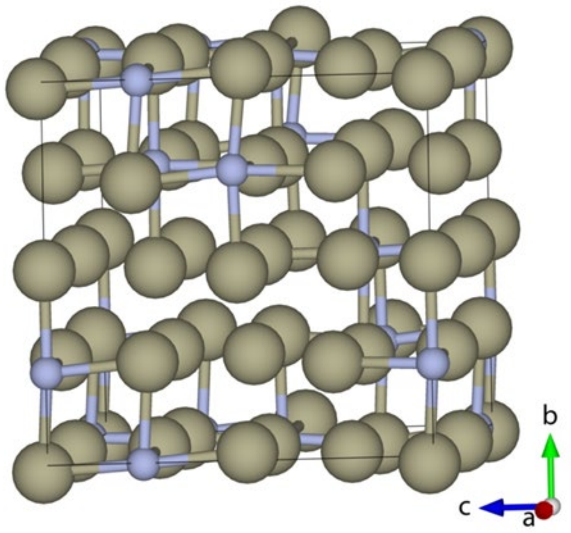

Supplementary Information Figure S9. Visualization of one exemplary $Re_{32}N_8$ supercell simulating Re-N cubic phase with NaCl (B1) type structure after structural relaxation.

Supplementary Information Tables.

Table S1. Summary of experiments

	Maximal pressure, GPa P1/P2*		Maximal temperature, K	Phases observed
	ref. 21	ref. 29		
dsDAC #1	1298(10)/173(3)	930(5)/149(3)	2200(200)	Re_7N_3, re-crystallized Re
dsDAC #2	974(2)/100(1)	646(2)/89(1)	2400(200)	Re_7N_3, re-crystallized Re
dsDAC #3	1132(7)/134(2)	730(4)/117(1)	3450(200)	ReN_x alloy, re-crystallized Re

*In each column the first value (P1) is the pressure on the sample, and the next value (P2) is the pressure on primary anvils (as determined from the Re EOS according to ref. 21 or ref. 29 (in ref. 29 the EOS of Re was measured in quasihydrostatic He pressure medium).

Table S2. Details of the crystal structure refinements of Re, Re_7N_3, and $ReN_{0.2}$ phases.

Pressure	1240(15)[21] or 905(5)[29] GPa		1132(7)[21] or 730(4)[29]
Chemical formula	Re	Re_7N_3	$ReN_{0.2}$
M_r	186.20	1345.43	189.01
Crystal system, space group	Hexagonal, $P6_3/mmc$	Hexagonal, $P6_3mc$	Cubic, *Fm-3m*
Temperature (K)	293	293	293
a, *c* (Å)	2.2269 (4),	6.2778 (19),	3.3994 (7)
c (Å)	3.5702 (15)	4.000 (2)	
V (Å^3)	15.33 (1)	136.53 (11)	39.28 (2)
Z	2	2	4
Radiation type (synchrotron)	λ = 0.30996 Å		0.2882 Å
μ (mm^{-1})	43.22	35.28	
Crystal size (mm)	0.001 × 0.001 × 0.001		
Diffractometer	ID11, ESRF		
Absorption correction	*CrysAlis PRO* 1.171.40.84a (Rigaku Oxford Diffraction, 2019) Spherical absorption correction using equivalent radius and absorption coefficient. Empirical absorption correction using spherical harmonics, implemented in SCALE3 ABSPACK scaling algorithm.		
T_{min}, T_{max}			
No. of measured, independent and observed [$I > 2\sigma(I)$] reflections	54, 20, 19	394, 196, 148	66, 17, 17
R_{int}	0.075	0.030	0.114
$(\sin \theta/\lambda)_{max}$ (Å^{-1})	0.875	0.954	1.130
$R[F^2 > 2\sigma(F^2)]$, $wR(F^2)$, S	0.086, 0.209, 1.34	0.057, 0.099, 1.09	0.050, 0.116, 1.54
No. of reflections	20	196	17
No. of parameters	2	12	1
$\Delta\rho_{max}$, $\Delta\rho_{min}$ (e Å^{-3})	8.01, −10.07	3.03, −3.22	9.03, −6.68

Table S3. Lattice parameters of Re and Re_7N_3 obtained during Le Bail fit of the powder diffraction pattern from dsDAC #2.

Phase	*a,* Å	*c*, Å	*V*, Å^3	P, GPa ref.[29]	P, GPa ref.[21]
Re LP	2.6120(2)	4.1942(6)	24.782(4)	89(1)	100(1)
Re HP	2.2945(3)	3.6972(8)	16.856(3)	646(2)	974(2)
Re_7N_3	6.3010(7)	4.0469(6)	139.15(2)		

Table S4. Crystallographic data for Re_7N_3 calculated at experimental volumes and temperature T=0 K.

	Re_7N_3,<br>*P*6_3*mc*<br>PBE
V, Å^3	136.5
Pressure, GPa	732
a, Å	6.2772
c, Å	4.0007
Fractional atomic coordinates (x/a, y/b, z/c)	Re1<br>(0.3333, 0.6667; 0.8094)<br>Re2<br>(0.4556; 0.54440; 0.2934)<br>Re3<br>(0.1223; 0.8777; 0.9831)<br>N<br>(0.1897; 0.8103; 0.5616)

Table S5. Formation enthalpies of 2×1×2 supercells with $Re_{16}N_x$ (x=2,3,4) stoichiometry with different occupations of octapores by N atoms simulating Re-N cubic phase with NaCl (B1) type structure at 650 GPa.

Concentration of N	Enthalpy of formation, eV/atom	
0.000000	0.000000	Re HCP
0.111111	-0.055177	$Re_{16}N_2$
0.111111	-0.073184	$Re_{16}N_2$
0.111111	-0.055169	$Re_{16}N_2$
0.111111	-0.064662	$Re_{16}N_2$
0.157895	-0.212287	$Re_{16}N_3$
0.157895	-0.227716	$Re_{16}N_3$
0.157895	-0.144791	$Re_{16}N_3$
0.157895	-0.227759	$Re_{16}N_3$
0.157895	-0.208550	$Re_{16}N_3$
0.157895	-0.227710	$Re_{16}N_3$
0.157895	-0.208550	$Re_{16}N_3$
0.200000	-0.389473	$Re_{16}N_4$
0.200000	-0.346507	$Re_{16}N_4$
0.200000	-0.389651	$Re_{16}N_4$
0.200000	-0.370255	$Re_{16}N_4$
0.200000	-0.383485	$Re_{16}N_4$
0.200000	-0.370427	$Re_{16}N_4$
0.200000	-0.383653	$Re_{16}N_4$
0.200000	-0.370337	$Re_{16}N_4$
0.200000	-0.389473	$Re_{16}N_4$
0.200000	-0.389489	$Re_{16}N_4$
0.200000	-0.324581	$Re_{16}N_4$
0.200000	-0.370428	$Re_{16}N_4$
1.000000	0.000000	cg-N

Table S6. Formation enthalpies of 2×1×2 supercells with $Re_{16}C_x$ (x=2,3,4) stoichiometry with different occupations of octapores by C atoms simulating Re-C cubic phase with NaCl (B1) type structure at 650 GPa.

Concentration of N	Enthalpy of formation, eV/atom	
0.000000	0.000000	Re HCP
0.111111	0.314061	$Re_{16}C_2$
0.111111	0.321492	$Re_{16}C_2$
0.111111	0.316490	$Re_{16}C_2$
0.157895	0.300479	$Re_{16}C_3$
0.157895	0.303620	$Re_{16}C_3$
0.157895	0.303597	$Re_{16}C_3$
0.157895	0.289469	$Re_{16}C_3$
0.157895	0.289494	$Re_{16}C_3$
0.157895	0.289495	$Re_{16}C_3$
0.200000	0.252995	$Re_{16}C_4$
0.200000	0.261154	$Re_{16}C_4$
0.200000	0.276412	$Re_{16}C_4$
0.200000	0.253007	$Re_{16}C_4$
0.200000	0.289973	$Re_{16}C_4$
0.200000	0.252827	$Re_{16}C_4$
0.200000	0.240137	$Re_{16}C_4$
0.200000	0.253007	$Re_{16}C_4$
0.200000	0.290011	$Re_{16}C_4$
0.200000	0.252822	$Re_{16}C_4$
1.000000	0.000000	Diamond-C

Table S7. Lattice parameters of the phases obtained during Le Bail fit of powder diffraction pattern from dsDAC #3.

Phase	*a*, Å	*c*, Å	*V*, Å^3	P, GPa (ref.[29])	P, GPa (ref.[21])
Re LP	2.5876(3)	4.123(4)	23.91(2)	117(1)	134(2)
Re HP	2.2803(3)	3.622(1)	16.31(2)	730(4)	1132(7)
$ReC_{0.6}$	2.7266(8)	4.469(3)	28.77(3)		
$ReN_{0.2}$	3.3994 (7)		39.28 (2)		

Supplementary references:

1. Blöchl, P. E. Projector augmented-wave method. *Phys. Rev. B* **50**, 17953–17979 (1994).
2. Kresse, G. & Joubert, D. From ultrasoft pseudopotentials to the projector augmented-wave method. *Phys. Rev. B* **59**, 1758–1775 (1999).
3. Kresse, G. & Furthmüller, J. Efficient iterative schemes for ab initio total-energy calculations using a plane-wave basis set. *Phys. Rev. B - Condens. Matter Mater. Phys.* **54**, 11169–11186 (1996).
4. Perdew, J. P., Burke, K. & Ernzerhof, M. Generalized Gradient Approximation Made Simple. *Phys. Rev. Lett.* **77**, 3865–3868 (1996).
5. Monkhorst, H. J. & Pack, J. D. Special points for Brillouin-zone integrations. *Phys. Rev. B* **13**, 5188–5192 (1976).
6. Methfessel, M. & Paxton, A. T. High-precision sampling for Brillouin-zone integration in metals. *Phys. Rev. B* **40**, 3616–3621 (1989).
7. Blöchl, P. E., Jepsen, O. & Andersen, O. K. Improved tetrahedron method for Brillouin-zone integrations. *Phys. Rev. B* **49**, 16223–16233 (1994).
8. Togo, A. & Tanaka, I. First principles phonon calculations in materials science. *Scr. Mater.* **108**, 1–5 (2015).
9. Cowley, R. A. Anharmonic crystals. *Reports Prog. Phys.* **31**, 303 (1968).
10. Friedrich, A. *et al.* Novel Rhenium Nitrides. Phys. Rev. Lett. **105**, 085504 (2010).
11. Hellman, O., Abrikosov, I. A. & Simak, S. I. Lattice dynamics of anharmonic solids from first principles. *Phys. Rev. B* **84**, 180301 (2011).
12. Hellman, O., Steneteg, P., Abrikosov, I. A. & Simak, S. I. Temperature dependent effective potential method for accurate free energy calculations of solids. *Phys. Rev. B - Condens. Matter Mater. Phys.* **87**, 1–8 (2013).
13. Hellman, O. & Abrikosov, I. A. Temperature-dependent effective third-order interatomic force constants from first principles. *Phys. Rev. B* **88**, 144301 (2013).

14. Giannozzi, P. *et al.* Quantum ESPRESSO toward the exascale. *J. Chem. Phys.* **152**, 154105 (2020).

15. Zhao, Z. *et al.* Nitrogen concentration driving the hardness of rhenium nitrides. *Sci. Rep.* **4**, 4797 (2015).

16. Zhao, Z. *et al.* Bulk Re_2C: Crystal structure, hardness, and ultra- incompressibility. *Cryst. Growth Des.* **10**, 5024–5026 (2010).

17. Bykov, M. *et al.* High-pressure synthesis of ultraincompressible hard rhenium nitride pernitride Re2(N2)(N2) stable at ambient conditions. *Nat. Commun.* **10**, 2994 (2019).

18. Bykov, M. *et al.* High-Pressure Synthesis of a Nitrogen-Rich Inclusion Compound $ReN_8 \cdot x N_2$ with Conjugated Polymeric Nitrogen Chains. *Angew. Chemie - Int. Ed.* **57**, 9048–9053 (2018).

19. Pettifor, D. F. *Bonding and Structure of Molecules and Solids*. (Clarendon Press, 1995).

20. Tidholm, J. *et al.* Temperature dependence of the Kohn anomaly in bcc Nb from first-principles self-consistent phonon calculations. *Phys. Rev. B* **101**, 115119 (2020).

21. Dubrovinsky, L., Dubrovinskaia, N., Prakapenka, V. B. & Abakumov, A. M. Implementation of micro-ball nanodiamond anvils for high-pressure studies above 6 Mbar. *Nat. Commun.* **3**, 1163–1167 (2012).

22. Dubrovinsky, L. *et al.* The most incompressible metal osmium at static pressures above 750 gigapascals. *Nature* **525**, 226–229 (2015).

23. Yokoo, M. *et al.* Ultrahigh-pressure scales for gold and platinum at pressures up to 550 GPa. *Phys. Rev. B* **80**, 104114 (2009).

24. Dubrovinskaia, N. *et al.* Terapascal static pressure generation with ultrahigh yield strength nanodiamond. *Sci. Adv.* **2**, e1600341 (2016).

25. Sakai, T. *et al.* High-pressure generation using double stage micro-paired diamond anvils shaped by focused ion beam. *Rev. Sci. Instrum.* **86**, 033905 (2015).

26. Lobanov, S. S. *et al.* Pressure, stress, and strain distribution in the double-stage diamond

anvil cell. *J. Appl. Phys.* **118**, (2015).

27. Vohra, Y. K. *et al.* High pressure studies using two-stage diamond micro-anvils grown by chemical vapor deposition. *High Press. Res.* **35**, 282–288 (2015).

28. Sakai, T. *et al.* High pressure generation using double-stage diamond anvil technique: problems and equations of state of rhenium. *High Press. Res.* **38**, 107–119 (2018).

29. Anzellini, S., Dewaele, A., Occelli, F., Loubeyre, P. & Mezouar, M. Equation of state of rhenium and application for ultra high pressure calibration. *J. Appl. Phys.* **115**, 043511 (2014).

30. Jenei, Z. *et al.* Single crystal toroidal diamond anvils for high pressure experiments beyond 5 megabar. *Nat. Commun.* **9**, 3563 (2018).

31. Dewaele, A., Loubeyre, P., Occelli, F., Marie, O. & Mezouar, M. Toroidal diamond anvil cell for detailed measurements under extreme static pressures. *Nat. Commun.* **9**, 2913 (2018).

32. Khandarkhaeva, S. *et al.* Novel Rhenium Carbides at 200 GPa. *Eur. J. Inorg. Chem.* **2020**, 2186–2190 (2020).
